# Intergroup contact throughout the lifespan modulates implicit racial biases across perceivers’ racial group

**DOI:** 10.1371/journal.pone.0180440

**Published:** 2017-07-11

**Authors:** Jennifer T. Kubota, Jaelyn Peiso, Kori Marcum, Jasmin Cloutier

**Affiliations:** 1 Department of Psychology, University of Chicago, Chicago, Illinois, United States of America; 2 Center for The Study of Race, Politics, and Culture, University of Chicago, Chicago, Illinois, United States of America; University of Bologna, ITALY

## Abstract

Few researchers have investigated how contact across the lifespan influences racial bias and whether diversity of contact is beneficial regardless of the race of the perceiver. This research aims to address these gaps in the literature with a focus on how diversity in childhood and current contact shapes implicit racial bias across perceivers’ racial group. In two investigations, participants completed an Implicit Association Test and a self-report measure of the racial diversity of their current and childhood contact. In both studies, increased contact with Black compared with White individuals, both in childhood (Study 2) and currently (Studies 1 and 2), was associated with reduced implicit pro-White racial bias. For Black individuals (Study 2) more contact with Black compared with White individuals also was associated with reduced implicit pro-White racial bias. These findings suggest that diversity in contact across the lifespan may be related to reductions in implicit racial biases and that this relationship may generalize across racial groups.

## Introduction

By 2020 more than half of the children in the United States will be minority and by 2044 the United States will become majority minority (U.S. Census Bureau, 2015). This greater population diversity will usher in an unprecedented era of intergroup contact in the United States. Social psychological research has long shown the benefits of intergroup contact, including reducing racial bias [1–11]. However, few researchers have investigated how contact across the lifespan influences racial bias and whether diversity of contact is beneficial regardless of the race of the individual. This research aims to address these gaps in the literature with a focus on how diversity in childhood and current contact shapes implicit racial attitudes across racial groups.

Although there is a growing body of literature exploring the relationship between intergroup contact and racial attitudes, most of this research has focused on explicit attitudes [12] with a small, but growing number of investigations exploring the impact of interracial contact on implicit associations [1,5,6,9–11,13]. Within these studies, the focus has been either on the impact of adult contact [14] or childhood contact [11,15–17] rather than on how contact across the lifespan shapes implicit racial bias.

Additionally, whereas most investigations of implicit racial bias reveal biases towards out-group members, research with adults and children has also identified implicit own-group bias (i.e. Black individuals with negative implicit Black attitudes) [11,18–20]. This form of own-group implicit bias may be the consequence of growing up in a cultural environment affording frequent exposure to negative associations about their own group [19,21,22]. One factor shaping the cultural impact on own-group implicit bias may be the extent to which members of minority racial groups have contact with majority outgroup individuals. However, scant research exists on own-group implicit bias, making it unclear whether own-group implicit bias is malleable and whether diversity in contact shapes implicit attitudes across diverse samples. Specifically, it is unclear whether greater diversity in contact increases or decreases own-group implicit racial bias, particularly for racial groups associated with negative stereotypes and prejudices.

In two studies, we examine how current (Study 1 and 2) and childhood contact (Study 2) with Blacks compared with Whites relates to implicit racial bias on the Implicit Association Task (IAT) [23] for a diverse sample (Study 1 and 2) and for Black individuals (Study 2). Based on previous theorizing about the role of culture in shaping implicit associations [19,21,22], we predicted that increased diversity in contact across the lifespan would decrease implicit racial bias across all participants and that more own-group contact would decrease negative implicit own-group associations for Black individuals (i.e., potentially less exposure to negative own-group associations).

## Study 1

### Materials and methods

#### Participants

University of Chicago human subjects research committee approved this research (IRB15-1559). The final sample included 396 participants (199 female; *M*_age_ = 34.67 years, *SD*_*age*_ = 11.231 years, *Min*_*age*_ = 18 years, *Max*_*age*_ = 68 years) collected via Amazon Mechanical Turk (MTurk, see “S1 File” for additional participant information). The sample was diverse (30 African American/Black, 23 Asian American, 4 Middle Eastern/Arab American, 306 White/Euro-American, 23 Latino/Hispanic American, 2 Native American, 8 Biracial/Multiracial). Post-hoc power analyses ensured that the final sample size was sufficiently powered to detect effects (see “S1 File” for a power analysis and exclusion criteria). All data and analysis scripts are available on Open Science Framework at https://osf.io/7bqng/.

#### Measures

Current contact questionnaire. Participants completed a questionnaire designed to measure current interracial contact [24]. Participants were asked to consider individuals in their immediate social networks and report the percentage of interracial contact, resulting in one averaged percentage for Blacks and one for Whites (see also “S1 File” for a list of all collected questionnaires). We then created an index of current interracial contact (Black Contact–White Contact).

Implicit association task (IAT). Implicit racial bias was assessed online with the IAT [23] via Inquisit (http://www.millisecond.com/download/library/iat/). IAT D scores were calculated using the procedures recommended by Greenwald, Nosek, and Banaji [25]. Scores can range from -2 to +2, with higher scores indicating a pro-White bias [19] (see “S1 File” for additional information).

#### Procedure

After consenting, participants completed the IAT (see “S1 File” for full Study 1 procedures) and then completed the current contact and demographics questionnaires.

### Results and discussion

#### Implicit racial bias

We initially explored the relationship between interracial contact and IAT. When comparing the participants’ IAT D-scores to 0, participants demonstrated pro-White implicit bias (*M*_*IATD*_ = .355, *SD* = .372, *t*(395) = 18.95, *p* < .001, *CI*_*lower*_ = .318 and *CI*_*upper*_ = .391).

#### Racial bias in current contact

Participants also had significant racial bias in current contact when comparing scores to 0, indicating less contact with Blacks than Whites (*M*_*current*_ = -52.495%, *SD* = 29.785%, *t*(395) = -35.07, *p* < .001, *CI*_*lower*_ = -55.437% and *CI*_*upper*_ = -49.552%).

#### Interracial current contact and implicit bias

**Fig 1:**
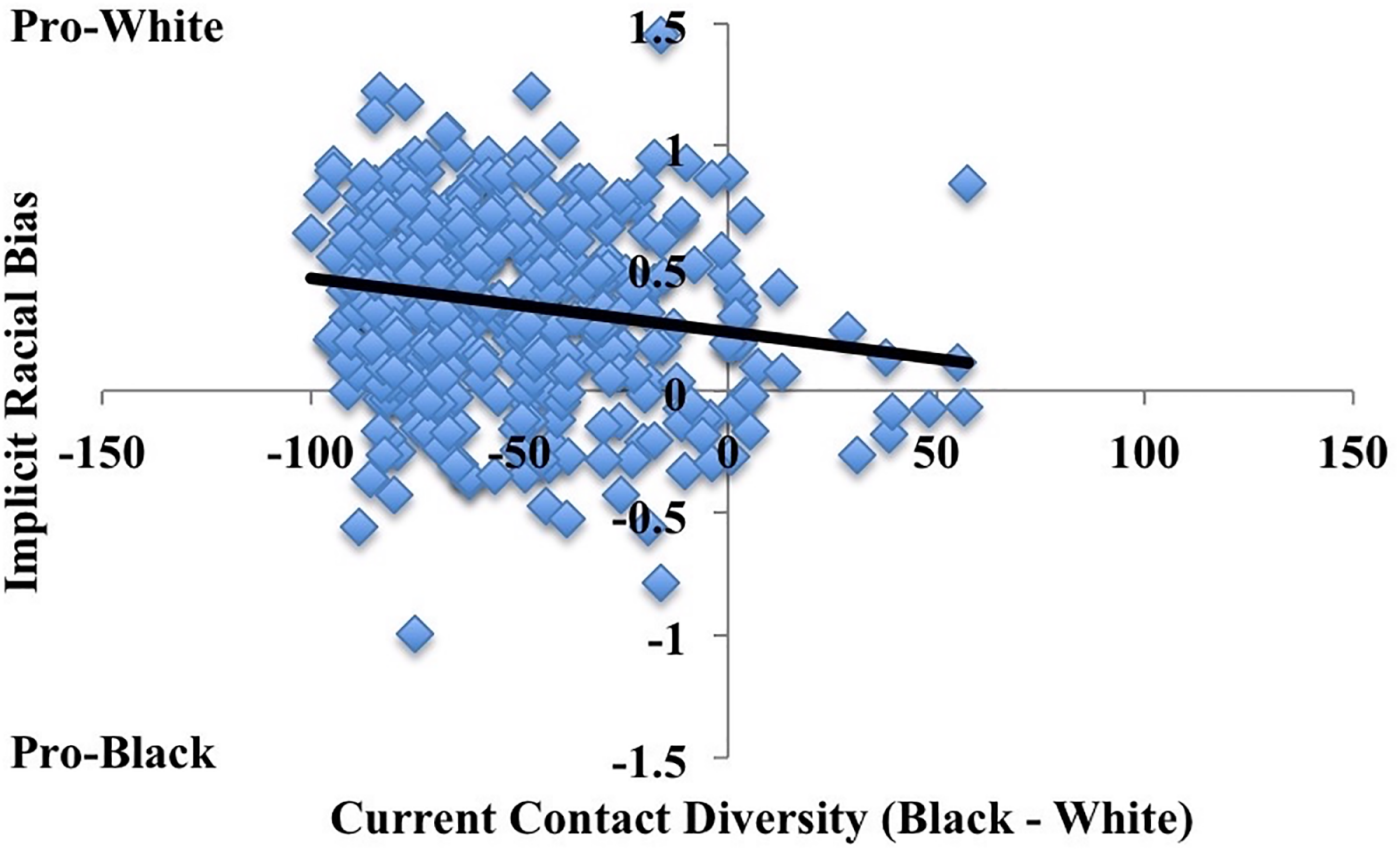
Relationship between current contact and implicit racial bias in Study 1. Higher values on implicit racial bias refer to more pro-White associations and lower numbers refer to more pro-Black associations. Overall, greater diversity in contact (Black contact–White contact) related to lower implicit racial bias (IAT D-scores).

Next, we explored the relationship between interracial current contact and implicit bias by regressing contact scores onto IATs. Replicating previous work, results revealed a significant main effect of current contact, *b* = -.002, *t*(395) = -3.537, *p*<0.001, *R*^*2*^ = .031, *CI*_*lower*_ = -0.003 and *CI*_*upper*_ = -0.001, such that greater current interracial contact related to lower implicit racial bias (Fig 1). Neither participant race nor the interaction between race and current contact emerged as significant predictors, indicating that the effects of contact on implicit racial bias did not differ across racial groups (see “S1 File” for analyses as function participant race). In fact, across the diverse, White, and non-White samples, greater interracial current contact was related to a reduction in implicit bias (see “S1 File” for additional analyses for Study 1).

## Study 2

To replicate and extend the findings of Study 1, we collected a diverse sample that included more Black participants and measured both childhood and current interracial contact.

### Materials and methods

#### Participants

The final sample included 367 participants (205 female, 161 male, and 1 failed to report; *M*_age_ = 33.79 years, *SD*_*age*_ = 10.66 years, *Min*_*age*_ = 18 years, *Max*_*age*_ = 72 years, 1 failed to report) recruited via MTurk and compensated $4 (see “S1 File” for additional participant information and exclusion criteria). The sample was diverse (100 African American/Black, 12 Asian American/Pacific Islander, 4 South East Asian, 220 Caucasian/Euro-American/White, 6 Latino, 6 Native American/American Indian, 19 Biracial/Multiracial). Post-hoc power analyses were conducted to ensure that the final sample size was sufficiently powered (see “S1 File” for a power analysis for Study 2).

#### Measures

Childhood and current contact questionnaires. For Study 2, participants completed a similar current contact measure as Study 1 and a childhood contact measure [26,27]. Participants were asked to consider individuals in their immediate social networks such as friends, caretakers, neighbors, and classmates, and report the percentage of interracial contact during four stages (before age 6, age 6 to 12, and age 13 to 18, and currently), resulting in four averaged percentages for Blacks and four for Whites. A childhood contact index was calculated by averaging all four stages (i.e. before age 6, age 6 to 12, and age 13 to 18) for Black and White contact and subtracting average White from average Black childhood contact (see “S1 File” for additional information regarding these questionnaires). Additionally, for exploratory analyses we separately investigated the impact of contact during the four measured periods of childhood (before age 6, age 6 to 12, and age 13 to 18) and created three separate indices by subtracting childhood contact during each stage with White individuals from Black individuals. Finally, a current contact index was calculated by subtracting averaged White from averaged Black current contact.

Implicit association task (IAT). The IAT was identical to Study 1.

#### Procedure

After reading a brief description of the study, participants were directed to the same online race IAT. After this task, participants completed the contact and demographics questionnaires.

### Results and discussion

#### Diverse participants

Implicit racial bias. On average, White participants typically have IAT scores above 0; however, other race participants have more D-score variability [19]. For example, 40% of Black Americans show pro-white associations, 40% shows pro-black associations, and 20% show no difference [19,28]. IAT variability is thought to reflect in part cultural learning of racial associations [19,21,22]. When comparing the diverse participants’ IAT D-scores to 0, participants demonstrated pro-White implicit bias (*M*_*IATD*_ = 0.294, *SD* = 0.425, *t*(366) = 13.243, *p*<0.001, *CI*_*lower*_ = 0.250 and *CI*_*upper*_ = 0.337).

Racial bias in childhood and current contact. Participants had significant racial bias in childhood contact when comparing scores to 0, indicating less childhood contact with Blacks than Whites (*M*_*childhood*_ = -36.646%, *SD* = 55.444%, *t*(366) = -12.662, *p*<0.001, *CI*_*lower*_ = -42.337% and *CI*_*upper*_ = -30.955%). This bias in contact was observed at each childhood period (before age 6: *M*_*childhood0-6*_ = -36.532%, *SD* = 60.766%, *t*(356) = -11.359, *p*<0.001, *CI*_*lower*_ = -42.857% and *CI*_*upper*_ = -30.208%; age 6 to 12: *M*_*childhood6-12*_ = -36.975%, *SD* = 56.745%, *t*(359) = -12.363, *p*<0.001, *CI*_*lower*_ = -42.856% and *CI*_*upper*_ = -31.093%; age 13 to 18: *M*_*childhood13-18*_ = -36.748%, *SD* = 51.369%, *t*(360) = -13.592, *p*<0.001, *CI*_*lower*_ = -42.065% and *CI*_*upper*_ = -31.431%). Participants also had significant racial bias in current contact when comparing scores to 0, indicating less current contact with Blacks than Whites (*M*_*current*_ = -36.234%, *SD* = 53.362%, *t*(366) = -13.008, *p*<0.001, *CI*_*lower*_ = -41.712% and *CI*_*upper*_ = -30.757%).

**Fig 2:**
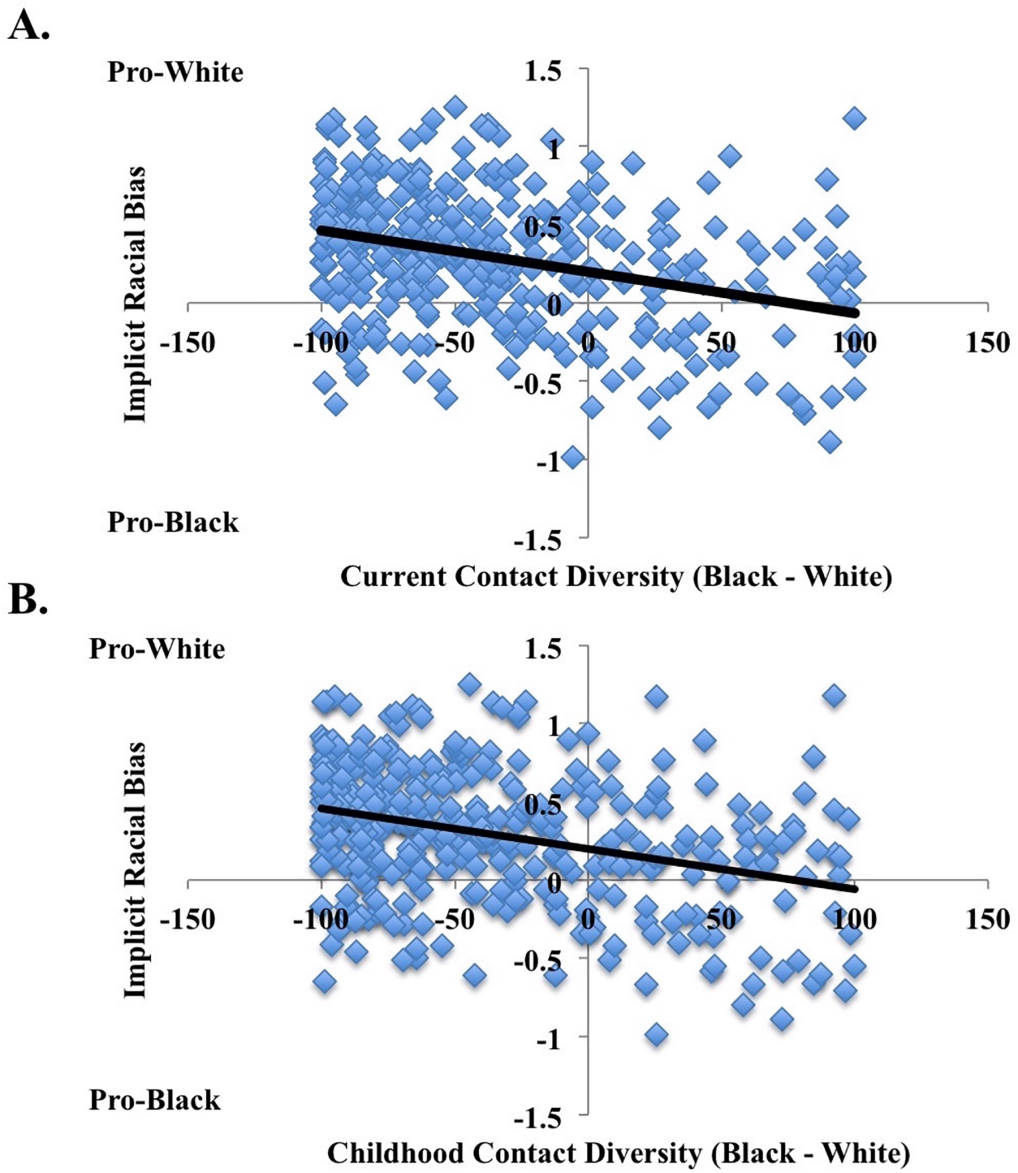
**Relationship between current (Panel A) and childhood contact (Panel B) and implicit racial bias in Study 2 for all participants**. Higher values on implicit racial bias refer to more pro-White associations and lower numbers refer to more pro-Black associations. Overall, greater diversity in contact (Black contact–White contact) related to lower implicit racial bias (IAT D-scores).

Interracial contact and implicit bias. For childhood contact, results revealed a significant main effect of childhood contact, *b* = -0.003, *t*(366) = -6.849, *p*<0.001, *R*^*2*^ = 0.114, *CI*_*lower*_ = -0.003 and *CI*_*upper*_ = -0.002, such that greater diversity in childhood contact related to less implicit racial bias (Fig 2). We next explored how childhood contact at each developmental period influenced implicit racial bias. Each childhood period separately revealed a significant main effect of contact (before age 6: *b* = -0.002, *t*(356) = -6.909, *p*<0.001, *R*^*2*^ = 0.119, *CI*_*lower*_ = -0.003 and *CI*_*upper*_ = -0.002; ages 6 to 12: *b* = -0.002, *t*(359) = -6.347, *p*<0.001, *R*^*2*^ = 0.101, *CI*_*lower*_ = -0.003 and *CI*_*upper*_ = -0.002; ages 13 to 18: *b* = -0.002, *t*(360) = -5.779, *p*<0.001, *R*^*2*^ = 0.085, *CI*_*lower*_ = -0.003 and *CI*_*upper*_ = -0.002), such that greater diversity in contact at each developmental period correlated with less implicit racial bias.

Next to explore whether any particular period of childhood contact has more explanatory power when predicting implicit racial bias, we regressed simultaneously childhood contact before age 6, childhood contact between 6 to 12 years of age, and childhood contact between 13 to 18 years of age on IATs scores. When all developmental periods were assessed in the model, only childhood contact before age 6 predicted implicit bias above and beyond the other developmental periods (b = -0.002, *t*(347) = -2.978, *p* = 0.003, *partial r*^*2*^ = 0.025, *CI*_*lower*_ = -0.004 and *CI*_*upper*_ = -0.001). Please note not all participants provided complete data for each childhood time period, reducing the degrees of freedom for these analyses. Because analyses of each childhood period were exploratory, participants were included in analyses of overall childhood contact if they reported at least one period of Black and White childhood contact. No other predictors remained significant (childhood contact between 6 to 12 years of age: b = -0.00006, *t*(347) = -.052, *p* = 0.959, *partial r*^*2*^ = 0.000009, *CI*_*lower*_ = -0.002 and *CI*_*upper*_ = 0.002; childhood contact between 13 to 18 years of age: b = 0.0002, *t*(347) = 0.164, *p* = 0.869, *partial r*^*2*^ = 0.00008, *CI*_*lower*_ = -0.002 and *CI*_*upper*_ = 0.002). These results indicate that self-reported childhood contact before age 6 may have more explanatory power in predicting decreases in implicit racial bias.

Additionally, replicating previous work and Study 1, linear regression results revealed a significant main effect of current contact, *b* = -0.003, *t*(366) = -6.729, *p*<0.001, *R*^*2*^ = 0.110, *CI*_*lower*_ = -0.003 and *CI*_*upper*_ = -0.002, such that greater diversity in current contact related to less implicit racial bias (Fig 2). Therefore, as childhood and current contact with Blacks relative to Whites increased, implicit racial bias decreased. Additionally, neither participant race nor the interaction between race and current or childhood contact emerged as significant predictors, indicating that the effects of contact on implicit racial bias did not differ across racial groups (see “S1 File” for additional analyses).

To explore whether racial bias in childhood or current interracial contact has more explanatory power when predicting implicit racial bias, we regressed childhood contact, current contact, and their interaction on IATs. Because childhood contact and current contact are highly correlated (*r*(365) = 0.876, *p* < .001) and may share a significant portion of explanatory variance, we report variance inflation factors for each predictor in our analyses. Predictors were initially simultaneously entered into a regression model. Current contact (VIF = 4.301), childhood contact (VIF = 4.470), and their interaction (VIF = 1.234) combined accounted for a small but significant 12.0% of variance in implicit bias, (*r*^*2*^ = 0.120, *F*_*change*_(3,363) = 16.456, *p* < .001). In the multiple regression model, childhood racial bias marginally predicted implicit bias above and beyond current racial contact and the interaction between childhood and current racial contact (b = -0.002, *t*(366) = -1.884, *p* = 0.060, *partial r*^*2*^ = 0.010, *CI*_*lower*_ = -0.003 and *CI*_*upper*_ = 0.00007). No other predictors remained significant (Current Contact: b = -0.001, *t*(366) = -1.535, *p* = 0.126, *CI*_*lower*_ = -0.003 and *CI*_*upper*_ = 0.0004; Childhood Contact*Current Contact: b = 0.000001, *t*(366) = 0.169, *p* = 0.866, *CI*_*lower*_ = -0.00001 and *CI*_*upper*_ = 0.00002). Predictors were then entered into a stepwise regression from the most distal (childhood) to most proximal contact (current) followed by the interaction. In the stepwise regression, including current contact as a predictor in the model did not account for a significant additional portion of variance (*r*^*2*^_*change*_ = 0.006, *F*_*change*_(1,364) = 2.383, *p* = 0.124) nor did including the interaction (*r*^*2*^_*change*_ = 0.00007, *F*_*change*_(1,363) = 0.029, *p* = 0.866). If instead the most proximal predictor is added first (current contact) into a stepwise regression, including childhood contact as a predictor in the model accounts for a marginally significant additional portion of variance (*r*^*2*^_*change*_ = 0.009, *F*_*change*_(1,364) = 3.837, *p* = 0.051).

In the full model where all factors were added simultaneously, the VIF factors indicated moderate amounts of multicollinearity. Therefore, we removed the interaction term and re-ran the analyses. When doing so, the overall model (*r*^*2*^ = 0.120, *F*_*change*_(2,364) = 24.736, *p*<0.001) was a significant fit, but only childhood contact remained marginally significant (childhood contact b = -0.002, *t*(366) = -1.959, *p* = 0.051, *partial r*^*2*^ = 0.010, *CI*_*lower*_ = -0.003 and *CI*_*upper*_ = 0.000006, VIF = 4.295 and current contact b = -0.001, *t*(366) = -1.544, *p* = 0.124, *partial r*^*2*^ = 0.007, *CI*_*lower*_ = -0.003 and *CI*_*upper*_ *=* 0.0003, VIF = 4.295). Therefore, although some of the relationship between childhood contact and implicit racial bias could be explained by current contact (although not significantly), childhood contact remained a marginally significant predictor when including current contact and the interaction between childhood contact and current contact. In addition, adding childhood contact to the model accounted for a marginally significant change in fit. This may imply that childhood contact is a slightly stronger predictor of implicit racial bias than current contact.

#### Black participants

Implicit racial bias. When comparing the participants’ IAT D-scores to 0, Black participants did not demonstrate implicit bias (*M*_*IATD*_ = 0.061, *SD* = 0.441, *t*(99) = 1.392, *p* = 0.167, *CI*_*lower*_ = -0.026 and *CI*_*upper*_ = 0.149).

Racial bias in contact. Black participants had significantly greater childhood contact with Blacks than Whites when comparing scores to 0 (*M*_*childhood*_ = 32.397%, *SD* = 42.490%, *t*(99) = 7.625, *p* < .001, *CI*_*lower*_ = 23.966% and *CI*_*upper*_ = 40.828%) and significantly greater current contact with Blacks than Whites when comparing scores to 0 (*M*_*current*_ = 25.666%, *SD* = 48.990%, *t*(99) = 5.239, *p* < .001, *CI*_*lower*_ = 15.946% and *CI*_*upper*_ = 35.387%). The tendency for Black participants to have more contact with Blacks than Whites was observed at each childhood period (before age 6: *M*_*childhood0-6*_ = 43.147%, *SD* = 39.830%, *t*(97) = 10.724, *p*<0.001, *CI*_*lower*_ = 35.162% and *CI*_*upper*_ = 51.133%; age 6 to 12: *M*_*childhood6-12*_ = 27.839%, *SD* = 52.614%, *t*(95) = 5.184, *p*<0.001, *CI*_*lower*_ = 17.178% and *CI*_*upper*_ = 38.499%; age 13 to 18: *M*_*childhood13-18*_ = 15.838%, *SD* = 51.355%, *t*(95) = 3.022, *p* = .003, *CI*_*lower*_ = 5.432% and *CI*_*upper*_ = 26.243%).

**Fig 3:**
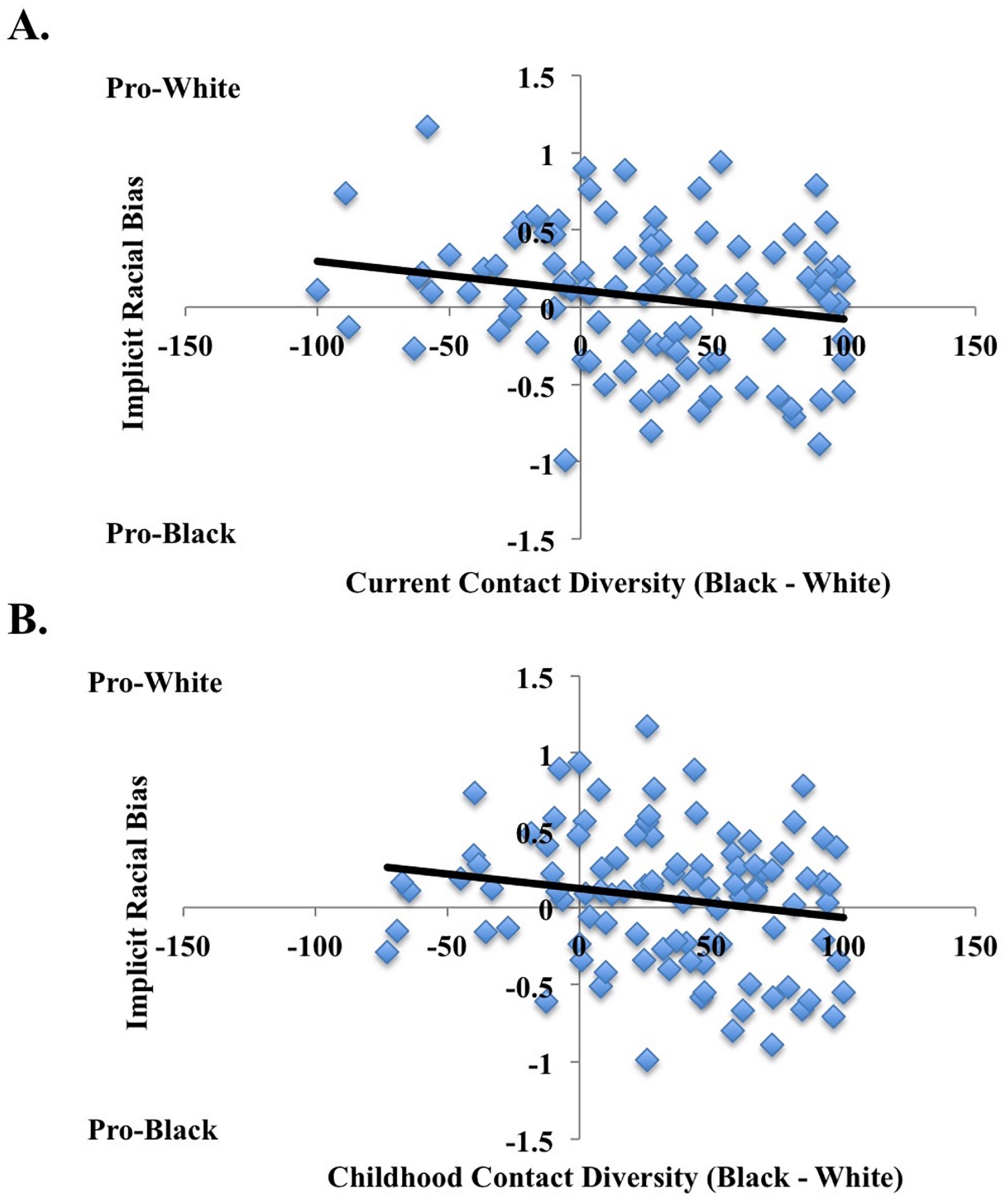
**Relationship between current (Panel A) and childhood contact (Panel B) and implicit racial bias in Study 2 for Black participants.** Higher values on implicit racial bias refer to more pro-White associations and lower numbers refer to more pro-Black associations. Overall, greater current ingroup contact (Black contact–White contact) related to lower own-group implicit racial bias (IAT D-scores).

Interracial contact and implicit bias. For Black participants, results did not reveal a significant main effect of overall childhood contact, *b* = -0.002, *t*(99) = -1.831, *p* = 0.070, *R*^*2*^ = 0.033, *CI*_*lower*_ = -0.004 and *CI*_*upper*_ = .0002) (see Fig 3). We next explored how childhood contact across developmental periods separately influenced implicit bias. In these analyses, only childhood contact between 6 to 12 years of age emerged as a significant predictor of implicit racial bias (*b* = -0.002, *t*(95) = -2.261, *p* = 0.026, *R*^*2*^ = 0.052, *CI*_*lower*_ = -0.004 and *CI*_*upper*_ = -0.0002), such that greater ingroup contact between ages 6 to 12 correlated with less implicit racial bias (before age 6: *b* = -0.002, *t*(97) = -1.498, *p* = 0.137, *R*^*2*^ = 0.023, *CI*_*lower*_ = -0.004 and *CI*_*upper*_ = 0.001; between 13 and 18 years old: *b* = -0.002, *t*(95) = -1.409, *p* = 0.162, *R*^*2*^ = 0.021, *CI*_*lower*_ = -0.003 and *CI*_*upper*_ = 0.0005).

Next to explore whether any particular period of childhood contact has more explanatory power when predicting implicit racial bias, we regressed childhood contact before age 6, childhood contact between 6 to 12 years of age, and childhood contact between 13 to 18 years of age on IATs scores. When all developmental periods were assessed simultaneously, no individual predictor emerged as significant (before age 6: b = -0.001, *t*(91) = .413, *p* = 0.680, *partial r*^*2*^ = 0.002, *CI*_*lower*_ = -0.003 and *CI*_*upper*_ = 0.004; between 6 to 12 years of age: b = -0.003, *t*(91) = -1.553, *p* = 0.124, *partial r*^*2*^ = 0.027, *CI*_*lower*_ = -0.007 and *CI*_*upper*_ = 0.001; between 13 to 18 years of age: b = 0.001, *t*(91) = 0.498, *p* = 0.620, *partial r*^*2*^ = 0.003, *CI*_*lower*_ = -0.003 and *CI*_*upper*_ = 0.004), indicating that accounting for all developmental time periods suppressed the significant bivariate relationship between childhood contact from 6 to 12 years of age and implicit racial bias.

Replicating previous work and the findings across all participants, results revealed a significant main effect of current contact, *b* = -0.002, *t*(99) = -2.102, *p* = 0.038, *R*^*2*^ = 0.043, *CI*_*lower*_ = -0.004 and *CI*_*upper*_ = -0.0001), such that the greater the individual’s current contact with Blacks compared with Whites, the lower their implicit racial bias (i.e., less pro-White associations) (see Fig 3). Therefore, as current contact with Blacks relative to Whites increased, pro-White associations decreased.

To explore whether racial bias in childhood or current interracial contact has more explanatory power when predicting implicit racial bias, we regressed childhood contact, current contact, and their interaction on IATs. Current contact (VIF = 2.271), childhood contact (VIF = 1.972), and their interaction (VIF = 1.948) combined were not a significant predictor of implicit bias for Black participants (*r*^*2*^_*change*_ = 0.049, *F*_*change*_(3,96) = 1.633, *p* = 0.187). In the context of the full model, none of the predictors were significant (all *ps* > .408). Although the VIF factors did not indicate multicollinearity, childhood contact and current contact were highly correlated (*r*(98) = 0.672, *p* < .001) and we wanted to be consistent in our analysis approach across all samples. Therefore, we removed the interaction term and re-ran the analyses. When doing so, the overall model was still not significant (*r*^*2*^_*change*_ = 0.046, *F*_*change*_(2,97) = 2.360, *p* = .100), and neither were the individual predictors (current contact b = -0.001, *t*(99) = -1.164, *p* = 0.247, *partial r*^*2*^ = .014, *CI*_*lower*_ = -0.004 and *CI*_*upper*_ = 0.001, VIF = 1.822; and childhood contact b = -0.001, *t*(99) = -0.577, *p* = 0.565, *partial r*^*2*^ = .003, *CI*_*lower*_ = -0.004 and *CI*_*upper*_ = 0.002, VIF = 1.822). Again, although some of the relationship between current contact and implicit racial bias could be explained by childhood contact for the Black sample, current contact when regressed on IAT scores alone was a predictor of implicit racial bias. In fact, including childhood contact as a predictor in the model did not account for a significant additional portion of variance (*r*^*2*^_*change*_ = 0.003, *F*_*change*_(1,97) = 0.333, *p* = 0565) nor did including the interaction (*r*^*2*^_*change*_ = 0.002, *F*_*change*_(1,96) = 0.218, *p* = .642). If instead the most distal predictor is added first (childhood contact) into the stepwise regression model as we did in all previous models first, including current contact as a predictor in the model does account for a significant additional portion of variance (*r*^*2*^_*change*_ = 0.013, *F*_*change*_(1,97) = 1.356, *p* = 0.247) in this sample.

For the diverse, non-White, non-Black, and Black participants the effects were directionally consistent (see “S1 File” for additional analyses), indicating that greater self-reported contact with Blacks compared with Whites related to decreased implicit pro-White bias. For the White and non-Black subsamples, contact was not a significant predictor of implicit racial bias (see “S1 File”). A reduction of power and variance (i.e., restriction of range) may have decreased our chances of finding a significant effect. Dropping Black participants effectively diminishes the IAT effect because Black participants have lower bias on the IAT and they give the correlation the full range of scores needed to detect the effect.

## Conclusions

Across two studies and racially diverse participants, self-reported contact, both current and during childhood, related to decreases in implicit racial bias. Across all participants, more contact with Blacks compared with Whites related to a decrease in implicit pro-White racial bias (Study 1 and 2) and for the Black participants (Study 2) more own-group current contact related to a decrease in negative implicit associations about Blacks (i.e., decreased implicit pro-White racial bias). The convergence of findings obtained from the diverse and Black only sample provides additional support for the theory that implicit bias may in part reflect stereotypical and/or prejudicial associative learning based on experience [29–32].

Nonetheless, different mechanisms may explain the observed relationship between self-reported contact across the lifespan and implicit racial bias across diverse participants. Indeed, contact has been proposed to impact implicit associations by influencing knowledge available about a social group, changing prejudicial behaviors, creating emotional bonds, changing intergroup anxiety, or by modifying ingroup associations [33–37]. For example, positive contact with Black individuals may provide accessible counterstereotypic examples that can contribute to reduced implicit bias [38]. This study did not assess these mechanisms and it remains unclear whether the mechanism(s) are conserved across racial groups. Moreover, greater conflict between the cultural stereotypes about their group and their personal experience may render minority groups’ own-group implicit associations more flexible. Future research should directly explore these different mechanisms across diverse racial groups.

At first glance the results of this study may seem to contradict research by Rae, Newheiser, & Olson [39]. Rae and colleagues observed that larger proportions of Black residents across U.S. states were associated with stronger implicit and in-group bias among both White and Black respondents. For White individuals, the greater the proportion of Black individuals across the state the greater their implicit pro-White associations. For Black individuals, the greater the proportion of Black individuals across the state the greater their implicit pro-Black associations. Therefore, we replicate the results with our Black participants. However, White participants data does not yield similar results. We speculate that the divergence in findings may be the result of measuring different kinds of contact. The present investigation assessed contact within social networks, which may be very different than the likelihood of encountering Black individuals in your environment for White participants. Future research should explore how contact quality versus quantity and environmental proximity of racial groups affects implicit associations across diverse populations.

In Study 2, current contact related to changes in implicit associations for Black participants whereas childhood and current contact related to changes in implicit associations when considering all participants in Study 1 and 2. Although this study was not designed to directly assess the mechanism(s) underlying these differences, early exposure to Black individuals may establish the scaffolding for challenging culturally transmitted stereotypes for White individuals. Additionally, although we did not observe a relationship between overall self-reported childhood contact and implicit associations for Black participants there was a relationship between self-reported early childhood contact between 6 and 12 years of age and implicit associations; and with increased power when assessing all participants even earlier self-reported contact, before age 6, was the best predictor of implicit associations. Therefore, childhood contact may be contributing to implicit associations for both groups but at different stages. Future research should explore how contact at different stages of the lifespan shape implicit associations.

Due to the correlational design of the study, causality cannot be inferred from the present research. Indeed, contact may reduce prejudice, but prejudice may also reduce contact [40]. Moreover, we used survey data to assess participants’ self-reported contact and early childhood memories may be inaccurately recalled. However, pervious research has found convergence between self-reported contact and the demographics of childhood residence based on zip code [27]. It may be the case that zip code information predicts social network contact in childhood to a better degree than state level data, but future should explore the relationship between childhood zip codes and state level demographics in predicting changes in implicit associations. Nonetheless, greater precision would be gained by exploring the relationship between contact and implicit associations in a longitudinal design. Moreover, because the focus of this research was on implicit associations, the current research did not assess explicit associations. Future research should strive to include explicit measures to explore how contact across the lifespan relates both to implicit and explicit associations.

In conclusion, the current study identified a small but significant relationship between self-reported contact and implicit biases among a diverse sample of the population and revealed a relationship between interracial contact and own-group bias among Black participants. Although both measures of childhood and current contact were included, they were strongly correlated and future research is needed to investigate their respective impact and the possibility of a critical period at which implicit bias solidifies during one’s lifetime. A better understanding of how contact impacts the formation and consolidation of implicit bias may prove central to the development of successful interventions to reduce implicit racial bias [41,42].

## Supporting information

S1 File Supplementary materials. This file contains supporting information regarding the methods, analyses, and additional discussion points for Study 1 and 2.
(DOCX)
