# Supplementary material for: Intergroup contact throughout the lifespan modulates implicit racial biases across perceivers’ racial group
Source: PLoS One. 2017 Jul 11;12(7):e0180440. doi: 10.1371/journal.pone.0180440 (PMC5507417; doi:10.1371/journal.pone.0180440)
Supplement: S1 File — This file contains supporting information regarding the methods, analyses, and additional discussion points for Study 1 and 2. (DOCX) [file pone.0180440.s001.docx]

**Supporting information**

***Study 1***

**Study 1 participants**

Four hundred and eighteen participants were recruited via Amazon’s Mechanical Turk (MTurk) who completed the study and were compensated $4. All U.S.-based workers at least 18 years of age with more than a 75% approval rating were eligible to participate. Of those consented, 22 respondents were not included in the final sample due to incomputable or incomplete D-scores (n = 16), or incomplete survey data (n = 6). The sample was diverse (30 African American/Black, 23 Asian American/Asian American, 4 Middle Eastern/Arab American, 306 White/Euro-American, 23 Latino/Hispanic American, 2 Native American, 8 Biracial/Multiracial), with 179 democrats, 74 republicans, 143 independents, and 87.6% self-identified as middle class or lower.

We a priori decided to collapse across both samples to explore contact in implicit attitudes, which was not the focus of initial data collection for the two studies about intergroup jury decision-making (see S3). We decided to combine these data prior to analyzing these contact data or exploring the relationship between IAT and contact. As these data were collected in the context of these other two studies, our a priori determined sample size was 200 for each study and data collection ceased after collecting 202 participants for the first study and 216 for the second study. The total sample size for the combined study was 416 participants. A post-hoc power analysis was conducted with G*Power 3.1 (<http://www.gpower.hhu.de/en.html>) to ensure that the final sample size was sufficiently powered to detect the relationship between contact and implicit bias with an alpha of .05 at the observed effect size of *Partial R^2^* <= .031. Results from a linear multiple regression: fixed model, single regression coefficient analysis suggest that the sample was well powered to detect the critical effect, 1 – *β* = 1.000.

**Amazon mechanical turk**

Amazon Mechanical Turk (MTurk) is an online research labor market where individuals participate in studies for small payments. Although differential attrition has been shown to introduce bias in between-subject design implemented in MTurk (Berinsky, Huber, & Lenz, 2012), numerous studies have established the reliability of MTurk data across many domains in psychology (Buhrmester, Kwang, & Gosling, 2011; Horton, Rand, & Zeckhauser, 2011; Mason & Suri, 2012; Paolacci, Chandler, & Ipeirotis, 2010; Rand, 2012). MTurk subjects do not differ significantly from undergraduates in terms of basic mathematical ability or attentiveness (Paolacci et al., 2010) and some established findings that require reaction times replicate (Crump, McDonnell, & Gureckis, 2013). Additionally, the MTurk population tends to be more diverse than undergraduate samples and generally representative of U.S. income distributions, with most reporting under $60,000 USD in maximum annual income (Mason & Suri, 2012).

**Jury decision-making task**

These data were collected in the context of two jury decision-making studies. These two studies examined how people make guilt decisions based on race and context information. After consenting participants read a brief description of the jury task and completed the short jury decision-making portion of the study where they read about a fictitious court case and made guilt decisions. Following this task, participants were directed to Inquisit to take the race IAT (http://www.millisecond.com/). After the IAT, participants completed the current contact questionnaire and demographics questions (e.g. gender, political affiliation, etc.). The study took about 60 minutes to complete. These studies were run within two months of each other. The first jury study recruited 202 participants and the second jury study recruited 216 participants. The focus of the jury studies was not on interracial contact, but contact was collected along with a battery of other questionnaires for future exploratory analyses. Therefore, the jury decision-making data are not reported here. In addition, the effects do not differ as a function of jury study (see below S6).

**Implicit association test**

IAT D scores is a measure of the difference in mean response latencies between prejudice congruent (i.e., Black/Bad are paired) and prejudice incongruent trials (i.e. Black/Good are paired) divided by the pooled standard deviation of all response latencies. The IAT was administered via Inquisit 4 Web (Version 4.0.9, Millisecond Software, Seattle, Washington).

**Current contact and demographics questionnaires**

We collected a variety of individual difference measures related to racial contact that were never analyzed. These measures were included as part of a contact questionnaire scale development project that was undergoing piloting. Only data about the estimates of the percentages of contact with Blacks and Whites were analyzed (questions CPW1, CPW2, CPW3, CPW4, CPB1, CPB2, CPB3, and CPB4 below) and information about participant race, age, gender, political ideology identification, and self-reported SES. We list the complete questionnaires below for interested readers. As in previous research (Cloutier, Li, & Correll, 2014; Cloutier, Li, Mišić, Correll, & Berman, in press), we averaged the current contact questions for Whites (CPW1, CPW2, CPW3, and CPW4) and averaged the current contact questions for Blacks (CPB1, CPB2, CPB3, and CPB4). Then, we subtracted White contact from Black contact to assess current diversity in contact (Black contact – White contact). Contact scores could range from 0 to 100, with 0 indicating that 0% of the participant’s contact was with that racial group and 100 meaning that 100% of the participant’s contact was with that racial group.

**Questionnaires study 1**

RCM Please respond to the following questions by clicking with your mouse. There are no right or wrong answers, and your responses will remain confidential.

CW1 How many close White friends do you have?

- 0 (1)
- 1-2 (2)
- 3-4 (3)
- 5-9 (4)
- 10 or more (5)

CW2 How many close White acquaintances do you have?

- 0 (1)
- 1-2 (2)
- 3-4 (3)
- 5-9 (4)
- 10 or more (5)

CW3 How many White people have you dated?

- 0 (1)
- 1-2 (2)
- 3-4 (3)
- 5-9 (4)
- 10 or more (5)

CB1 How many close Black friends do you have?

- 0 (1)
- 1-2 (2)
- 3-4 (3)
- 5-9 (4)
- 10 or more (5)

CB2 How many close Black acquaintances do you have?

- 0 (1)
- 1-2 (2)
- 3-4 (3)
- 5-9 (4)
- 10 or more (5)

CB3 How many Black people have you dated?

- 0 (1)
- 1-2 (2)
- 3-4 (3)
- 5-9 (4)
- 10 or more (5)

CE1 In what type of environment have you spent most of your life?

- Urban (1)
- Suburban (2)
- Rural (3)

CE2 Would you describe the environment in which you have spent most of your life as racially diverse?

- Yes (1)
- No (2)

CE3 Would you describe the environment in which you have spent most of your life as culturally diverse?

- Yes (1)
- No (2)

CP For the next several items, you will be asked to make percentage ratings. For each item, please select a whole number (i.e., without decimals) between 0 and 100.Slide the bars using your mouse across the grid to indicate a percentage.  The number to the right of the scale indicates your chosen percentage.

CPW1

______ Approximately what percentage of all your close friends are White? (1)

CPW2

______ Approximately what percentage of all your acquaintances are White? (1)

CPW3

______ Approximately what percentage of the people you encounter on a day-to-day basis, in your neighborhood and at work or school, are White? (1)

CPW4

______ Approximately what percentage of the people you see in the media, including television, movies, magazines, and sports, are White? (1)

CPB1

______ Approximately what percentage of all your close friends are Black? (1)

CPB2

______ Approximately what percentage of all your acquaintances are Black? (1)

CPB3

______ Approximately what percentage of the people you encounter on a day-to-day basis, in your neighborhood and at work or school, are Black? (1)

CPB4

______ Approximately what percentage of the people you see in the media, including television, movies, magazines, and sports, are Black? (1)

Age Age ________

Gender Gender

- Male (1)
- Female (2)

Race Which ethnic group do you belong to?

- Black/African American (1)
- Asian/Asian American (2)
- White/Euro-American (3)
- Latino/Hispanic American (4)
- Middle Eastern/Arab American (5)
- Native American (6)
- Biracial/Multiracial (7) ____________________
- Other (please specify) (8) ____________________

Political What political party do you belong to or associate yourself with?

- Democrat (1)
- Republican (2)
- Independent (3)

School What type of high school did you attend?"

- General public (1)
- Specialized or magnet public (2)
- Private (3)
- Parochial or religious (4)

SES How would you describe your family's social class position?

- Poor (1)
- Working Class (2)
- Middle Class (3)
- Upper Middle Class (4)
- Upper Class (5)

Education What is the highest level of education you have completed?

- No Formal Education (1)
- Elementary School (2)
- Some High School (3)
- Completed High School (4)
- Some College (5)
- BA/BS Degree (6)
- Some Graduate/Professional School (7)
- Hold Graduate/Professional Degree (8)

Pol2 How would you describe your political party preference?

- Strong Republican (1)
- Weak Republican (2)
- Independent Republican (4)
- Independent (5)
- Independent Democrat (6)
- Weak Democrat (7)
- Strong Democrat (8)

PE In terms of economic issues, how would you describe your political attitudes and beliefs?

- Very Conservative (1)
- Conservative (2)
- Slightly Conservative (3)
- Middle-of-the-road (4)
- Slightly Liberal (5)
- Liberal (6)
- Very Liberal (7)
- Other (please specify) (8) ____________________

PS In terms of social issues, how would you describe your political attitude and beliefs?

- Very Conservative (1)
- Conservative (2)
- Slightly Conservative (3)
- Middle-of-the-road (4)
- Slightly Liberal (5)
- Liberal (6)
- Very Liberal (7)
- Other (please specify) (8) ____________________

Language: What is your native language?

Country: What country were you born in?

Years: If you were born in a country other than the United States, how long have you lived in the U.S?  If you were born in the United States, please go on to the next question.

**Study 1 additional results**

**Implicit bias and interracial contact for all participants**

In the context of the model exploring the relationship between interracial current contact and implicit bias, IAT scores were significantly different from 0 when controlling for interracial current contact (b_o_=0.239, *t*(395) = 6.402, *p*<0.001, *CI_lower_*=0.166 and *CI_upper_*=0.313).

We also regressed participant race (coded 1 for White and -1 for non-White), current interracial contact, and their interaction on implicit racial bias. Linear regression results revealed a significant main effect of current contact, b=-0.003, *t*(395)=-3.555, *p*<0.001, *partial r^2^*= 0.031, *CI_lower_*=-0.004 and *CI_upper_*=-0.001), such that greater interracial current contact was related to a reduction in implicit bias. IAT scores were significantly different from 0 when controlling for interracial current contact, participant race, and their interaction (B_o_=0.242, *t*(395) = 6.428, *p*<0.001, *CI_lower_*=0.168 and *CI_upper_*=0.316). All other predictors were non-significant: Participant Race, b=0.019, *t*(395)=0.497, *p*=0.619, *partial r^2^*= 0.0006, *CI_lower_*=-0.055 and *CI_upper_*=0.093; Participant Race*Current Contact, b=0.001, *t*(395)=1.045, *p*=0.297, *partial r^2^*=0.003, *CI_lower_*=-0.001 and *CI_upper_*=0.002).

**Implicit bias and interracial contact for white participants**

Although participant race did not interact with contact, we proceeded to verify the effects within our smaller samples of White participants and non-White participants. First, we explored the relationship between interracial contact and IAT for the White sample. When comparing the participants’ IAT D-scores to 0, participants demonstrated pro-White implicit bias (*M_IATD_*=0.365, *SD*=0.372, *t*(305)=17.249, *p*<0.001, *CI_lower_*=0.324 and *CI_upper_*=0.407). Participants also had significant racial bias in current contact when comparing scores to 0, indicating less contact with Blacks than Whites (*M_current_*= -59.260%, *SD*=24.243%, *t*(305)=-42.762, *p*<0.001, *CI_lower_*=-61.989% and *CI_upper_*=-56.535%).

Next, we explored the relationship between interracial current contact and implicit bias by regressing contact scores onto IATs. Replicating previous work and our findings from the whole sample, linear regression results revealed a small but significant main effect of current contact, b=-0.002, *t*(305)=-2.032, *p*=0.043, *R^2^*=0.013, *CI_lower_*=-0.003 and *CI_upper_*=-0.00006), such that greater interracial current contact was related to a reduction in implicit bias. IAT scores remained significantly different from 0 when controlling for interracial current contact (b_o_=0.260, *t*(305) = 4.674, *p*<0.001, *CI_lower_*=0.151 and *CI_upper_*=0.370).

**Implicit bias and interracial contact for non-white participants**

We then explored the relationship between interracial contact and IAT for the non-White sample. When comparing the participants’ IAT D-scores to 0, participants demonstrated pro-White implicit bias (*M_IATD_*=0.319, *SD*=0.379, *t*(89)=7.980, *p*<0.001, *CI_lower_*=0.239 and *CI_upper_*=0.398). Participants also had significant racial bias in current contact when comparing scores to 0, indicating less contact with Blacks than Whites (*M_current_*= -29.486%, *SD*=35.078%, *t*(89)=-7.974, *p*<0.001, *CI_lower_*=-36.833% and *CI_upper_*=-22.139%).

Next, we explored the relationship between interracial current contact and implicit bias by regressing contact scores onto IATs. Replicating the findings from the whole sample and the White sample, linear regression results revealed a significant main effect of current contact, b=-0.003, *t*(89)=-2.952, *p*=0.004, *R^2^*=0.090, *CI_lower_*= -0.005 and *CI_upper_*=-0.001), such that greater interracial current contact was related to a reduction in implicit bias. IAT scores remained significantly different from 0 when controlling for interracial current contact (b_o_=0.223, *t*(89) = 4.448, *p*<0.001, *CI_lower_*=0.123 and *CI_upper_*=0.323).

**Implicit bias and interracial contact for all participants as a function of jury study**

To ensure that our samples did not differ between the two jury studies, we explored whether IAT scores, contact, and the relationship between contact and IAT scores differed between the two groups. In the regression analyses, study was coded such that jury study 1=1 and jury study 2 =-1.

IAT scores did not differ as a function of the jury study across all participants (b=-0.004, *t*(395)=-0.225, *p*=0.822, *R^2^*=0.0001, *CI_lower_*= -0.041 and *CI_upper_*=0.033). In the context of the model exploring the relationship between study and implicit bias, IAT scores were significantly different from 0 when controlling for interracial current contact (*M*_IAT Study 1_ =0.3503, *SD*_IAT Study 1_ =0.3744 and *M*_IAT Study 2_ =0.359, *SD*_IAT Study 2_ =0.3711; b_o_=0.355, *t*(395)=18.919, *p*<0.001, *CI_lower_*=0.318 and *CI_upper_*=0.391).

Current contact did differ as a function of the jury study across all participants (b=-8.445, *t*(395)=-5.875, *p*<0.001, *R^2^*=0.081, *CI_lower_*=-11.271 and *CI_upper_*=-5.619). In the context of the model exploring the relationship between study and implicit bias, IAT scores were significantly different from 0 when controlling for interracial current contact (*M*_contact Study 1_ =-61.153, *SD*_contact Study 1_ =26.404 and *M*_contact Study 2_ =-44.263, *SD*_contact Study 2_ =30.535; b_o_=-52.708, *t*(395)=-36.667, *p*<0.001, *CI_lower_*=-55.534 and *CI_upper_*=-49.882).

Importantly, the effect of contact on IAT scores did not differ as a function of study (Current Contact: b=-0.002, *t*(395)=-3.807, *p*<0.001, *partial R^2^*=0.036, *CI_lower_*= -0.004 and *CI_upper_*=-0.001; Study: b=-0.047, *t*(395)=-1.155, *p*=0.249, *partial R^2^*=0.003, *CI_lower_*= -0.126 and *CI_upper_*=0.033; Study*Contact: b=-0.0004, *t*(395)=-0.618, *p*=0.537, *partial R^2^*=0.001, *CI_lower_*= -0.002 and *CI_upper_*=0.001).

**Study 2**

**Study 2 participants**

Four hundred thirty participants were recruited via MTurk and compensated $4. All U.S.-based workers at least 18 years of age with more than a 65% approval rating were eligible to participate. Of those consented, 47 respondents were not included in the final sample due to computer malfunctions that resulted in failure to register the respondents’ IDs (n = 14), incomputable D-scores (n = 13), outlier D-scores (n=1, *z*=-4.18), incomplete survey data (n = 27), or reporting discrepancies in their race (n=8; e.g. identifying as Black in the first part of the experiment and then identifying as White in the second part of the experiment). The final sample included 367 participants (205 female, 161 male, and 1 failed to report; *M*_age_ = 33.79 years, *SD_age_* = 10.66 years, *Min_age_* = 18 years, *Max_age_* = 72 years, 1 did not report). The sample was diverse (100 African American/Black, 12 Asian American/Pacific Islander, 4 South East Asian, 220 Caucasian/Euro-American/White, 6 Latino, 6 Native American/American Indian, 19 Biracial/Multiracial), with 173 democrats, 65 republicans, 127 independents (2 did not respond), and 86.6% identified as middle class or lower. Participants who were coded as White had to identify as White and non-Black and Black participants had to identify as Black and non-White or Asian.

As this was the first study to examine the impact of childhood and current contact on implicit attitudes in a diverse sample, we opted to recruit a larger sample compared to previous work (e.g., Aberson & Haag, 2007; White participants *N* = 153, minority participants = 0) and a similar sample size as Study 1. The data were collected in two phases. The first portion of data was collected as part of a master’s project and included 100 White participants. To increase sample diversity a second wave of data were collected within a few months of the first wave and included the following two collection criteria 1) at least 100 participants had to self-report their race as Black and 2) the final sample size across the waves for this study should be similar to Study 1 (n=400). Our data collection ceased after collecting 416 participants who provided usable respondent IDs, slightly above our intended sample. A post-hoc power analysis was conducted with G*Power 3.1 (<http://www.gpower.hhu.de/en.html>) to ensure that the final sample size was sufficiently powered to detect effects with an alpha of .05 at the observed effect size of *Partial R^2^* <=.114 for childhood contact. Results from a linear multiple regression: fixed model, single regression coefficient analysis suggest that the sample was well powered to detect the critical effects, 1 – *β* = 1.000. The final sample size was also sufficiently powered to detect effects with an alpha of .05 at the observed effect size of *Partial R^2^* <=.110 for current contact. Results suggest that the sample was well powered to detect the critical effects, 1 – *β* = 1.000.

**Current contact, childhood contact, and demographics questionnaires**

We collected a variety of individual difference measures related to racial contact that were never analyzed. These measures were included as part of a contact questionnaire scale development project that was undergoing piloting. Only data about the estimates of the percentages of contact with Blacks and Whites and information about participant race, age, gender, political ideology identification, and self-reported SES were analyzed. We list the complete questionnaires below for interested readers. Contact scores could range from 0 to 100, with 0 indicating that 0% of the participant’s contact was with that racial group and 100 meaning that 100% of the participant’s contact was with that racial group. As in previous research (Cloutier, Li, & Correll, 2014; Cloutier, Li, Mišić, Correll, & Berman, in press), we averaged the current contact questions for Whites (Q453_4_TEXT, Q454_4_TEXT, and Q141_4_TEXT) and averaged the current contact questions for Blacks (Q453_2_TEXT, Q454_2_TEXT, and Q141_2_TEXT). Then, we averaged the childhood contact questions for Whites (Q75_4_TEXT, Q79_4_TEXT, Q78_4_TEXT, Q81_4_TEXT, Q414_4_TEXT, Q415_4_TEXT, Q435_4_TEXT, and Q436_4_TEXT) and averaged the childhood contact questions for Blacks (Q75_2_TEXT, Q79_2_TEXT, Q78_2_TEXT, Q81_2_TEXT, Q414_2_TEXT, Q415_2_TEXT, Q435_2_TEXT, and Q436_2_TEXT). We also averaged the childhood contact questions for Whites from ages 0 to 6 (Q75_4_TEXT, Q79_4_TEXT, Q78_4_TEXT, Q81_4_TEXT); from ages 6 to 12 (Q414_4_TEXT, Q415_4_TEXT) and from ages 13 to 18 (Q435_4_TEXT, Q436_4_TEXT). We also averaged the childhood contact questions for Blacks from ages 0 to 6 (Q75_2_TEXT, Q79_2_TEXT, Q78_2_TEXT, Q81_2_TEXT); from ages 6 to 12 (Q414_2_TEXT, Q415_2_TEXT) and from ages 13 to 18 (Q435_2_TEXT, Q436_2_TEXT). Interracial childhood contact scores were then calculated by subtracting average childhood contact with Whites from average childhood contact with Blacks (Black – White) for overall childhood contact and separately for each childhood time period. Because analyses of each childhood period were exploratory, participants were included in analyses of overall childhood contact if they reported at least one period of Black and White childhood contact. Interracial current contact scores were also calculated by subtracting current contact with Whites from current contact with Blacks (Black – White). The study took about 45 minutes to complete.

**Questionnaires study 2**

Q53 Please take a moment to consider your childhood. Recall your earliest memories, think about pictures from photo albums, stories you have heard from your family, etc. We are interested in the people you saw regularly before the age of 6. This is roughly the period just when you started and before you went to elementary school. These people may include:-Family who lived nearby and  saw you regularly (at least once a month).-Friends of the family.-Babysitters and other caretakers.-People who worked in your house (such as caretakers).-Neighbors.-Other children - playmates, classmates in daycare/preschool.-Anyone else with whom you spent time.

Q55 List some of the people you remember before the age of 6. Name as many people in each category as you can (first names only). Try to go beyond the easy ones!

Friends of the family: (1)

Babysitters: (2)

Housekeepers (if any): (5)

Neighbors: (6)

Playmates: (3)

Others: (4)

Q67 What was your ZIP code growing up?

Q68 If you moved frequently, please indicate your ZIP codes below:

Q73 Please try to report as much as you can remember. If there is information that you do not remember, you may skip the question but please do not make up an answer.

Q74 Thinking about your childhood before age 6, please answer the following questions to the best of your ability.

Q75 1. What percentage of your family belonged to each of the following categories?

% Asian (1)

% Black (2)

% Latino (3)

% White (4)

% Other (5)

Q79 2. Not including your family, what percentage of the adults you knew (friends, caretakers, neighbors) belonged to each of the following categories?

% Asian (1)

% Black (2)

% Latino (3)

% White (4)

% Other (5)

Q78 3. Not including your family, what percentage of the children you knew (friends, classmates) belonged to each of the following categories?

% Asian (1)

% Black (2)

% Latino (3)

% White (4)

% Other (5)

Q81 4. Think about the supermarket where your family shopped. What percentage of the people who shopped at the supermarket belonged to each of the following categories?

% Asian (1)

% Black (2)

% Latino (3)

% White (4)

% Other (5)

Q82 5. If you used the "Other" category, please define the group(s) in the space below:

Other = (1)

Q84 Please click on the circle under the number that best corresponds with your answer to the question.

|  | 1 (1) | 2 (2) | 3 (3) | 4 (4) | 5 (5) | 6 (6) | 7 (7) |
| --- | --- | --- | --- | --- | --- | --- | --- |
| How confident are you about the answers you gave for questions 1-4? (2) |  |  |  |  |  |  |  |

Q85 Please click on the circle under the number that best corresponds with your answer to the question.

|  | 1 (1) | 2 (2) | 3 (3) | 4 (4) | 5 (5) | 6 (6) | 7 (7) |
| --- | --- | --- | --- | --- | --- | --- | --- |
| How much would you say you remember about your childhood before the age of 6? (1) |  |  |  |  |  |  |  |

Q86 During your early childhood (age 0 to 6), did you live in more than one town or city?

- Yes (5)
- No (6)

Q87 In what town did you spend most of your early childhood (age 0 to 6)? If you lived in more than one place, please list the two places where you have spent the most time as accurately as you can:

Q88 Describe the first town or city:

1st town or city: (1)

State (2)

ZIP code (3)

Answer If During your early childhood (age 0 to 6), did you live in more than one town or city? Yes Is Selected

Q90 Describe the second town or city

2nd town or city: (1)

State (2)

ZIP code (3)

Answer If During your early childhood (age 0 to 6), did you live in more than one town or city? Yes Is Selected

Q89 In what town did you spend most of your early childhood (age 0 to 6)?

Town or city: (1)

State (2)

ZIP code (3)

Q91 Negative interracial relations refer to situations where the relationships among individuals from different races tend to be tense, and people tend to avoid going to areas where racial out-group members gather. Positive interracial relations refer to situations where interactions tend to be pleasant and harmonious.

Answer If During your early childhood (age 0 to 6), did you live in more than one town or city? Yes Is Not Selected

Q92 Consider your life before age 6, how do you rate the quality of interracial relations in the neighborhood that you lived?

Answer If During your early childhood (age 0 to 6), did you live in more than one town or city? Yes Is Not Selected

Q93 Town or city you lived:

|  | 1 (1) | 2 (2) | 3 (3) | 4 (4) | 5 (5) | 6 (6) | 7 (7) |
| --- | --- | --- | --- | --- | --- | --- | --- |
| How would you rate the quality of interracial relationships in the town where you lived? (1) |  |  |  |  |  |  |  |

Answer If During your early childhood (age 0 to 6), did you live in more than one town or city? Yes Is Selected

Q94  Consider your life before age 6, how do you rate the quality of interracial relations in the neighborhoods that you lived? If you lived in more than one place, please report the average quality of interracial relations in all neighborhoods.

Answer If During your early childhood (age 0 to 6), did you live in more than one town or city? Yes Is Selected

Q95 Towns or cities you lived:

|  | 1 (1) | 2 (2) | 3 (3) | 4 (4) | 5 (5) | 6 (6) | 7 (7) |
| --- | --- | --- | --- | --- | --- | --- | --- |
| How would you rate the average quality of interracial relationships in the towns where you lived? (1) |  |  |  |  |  |  |  |

Q96 Please click on the circle under the number that best corresponds with your answer to the question.

|  | 1 (1) | 2 (2) | 3 (3) | 4 (4) | 5 (5) | 6 (6) | 7 (7) |
| --- | --- | --- | --- | --- | --- | --- | --- |
| Based on your observation of individuals living in the same neighborhood as you, did people interact with others within their own race? (1) |  |  |  |  |  |  |  |

Q97 Please click on the circle under the number that best corresponds with your answer to the question.

|  | 1 (1) | 2 (2) | 3 (3) | 4 (4) | 5 (5) | 6 (6) | 7 (7) |
| --- | --- | --- | --- | --- | --- | --- | --- |
| Based on your observation of individuals living in the same neighborhood as you, did people interact with others outside their race? (1) |  |  |  |  |  |  |  |

Q98 Please click on the circle under the number that best corresponds with your answer to the question.

|  | 1 (1) | 2 (2) | 3 (3) | 4 (4) | 5 (5) | 6 (6) | 7 (7) |
| --- | --- | --- | --- | --- | --- | --- | --- |
| Racial segregation, refers to the separation of individuals by race, may be seen in schools, public transportations, and housing. How would you rate the racial segregation in the neighborhood that you lived? (1) |  |  |  |  |  |  |  |

Q411 Please try to report as much as you can remember. If there is information that you do not remember, you may skip the question but please do not make up an answer.

Q412 Now consider your childhood between the ages of 6 and 12. This is roughly the time during which you were in elementary school. Please answer the following questions to the best of your ability.

Q414 1. Not including your family, what percentage of the adults you knew (friends, caretakers, neighbors) belonged to each of the following categories?

% Asian (1)

% Black (2)

% Latino (3)

% White (4)

% Other (5)

Q415 2. Not including your family, what percentage of the children you knew (friends, classmates, neighbors) belonged to each of the following categories?

% Asian (1)

% Black (2)

% Latino (3)

% White (4)

% Other (5)

Q141 Please type the number 5 into the box below.

Q418 Please click on the circle under the number that best corresponds with your answer to the question.

|  | 1 (1) | 2 (2) | 3 (3) | 4 (4) | 5 (5) | 6 (6) | 7 (7) |
| --- | --- | --- | --- | --- | --- | --- | --- |
| How confident are you about the answers you gave for questions 1 & 2? (1) |  |  |  |  |  |  |  |

Q419 Please click on the circle under the number that best corresponds with your answer to the question.

|  | 1 (1) | 2 (2) | 3 (3) | 4 (4) | 5 (5) | 6 (6) | 7 (7) |
| --- | --- | --- | --- | --- | --- | --- | --- |
| How much would you say you remember about your childhood from age 6 to 12? (1) |  |  |  |  |  |  |  |

Q420 During your late childhood (age 6 to 12), did you live in more than one town or city?

- Yes (5)
- No (6)

Q421 In what town did you spend most of your late childhood (age 6 to 12)? If you lived in more than one place, please list the two places where you have spent the most time as accurately as you can:

Q422 Please describe the first town or city:

1st town or city: (1)

State (2)

ZIP code (3)

Answer If During your late childhood (age 6 to 12), did you live in more than one town or city? Yes Is Selected

Q423 Please describe the second town or city:

2nd town or city: (1)

State (2)

ZIP code (3)

Q425 Negative interracial relations refer to situations where the relationships among individuals from different races tend to be tense, and people tend to avoid going to areas where racial out-group members gather. Positive interracial relations refer to situations where interactions tend to be pleasant and harmonious.

Answer If During your late childhood (age 6 to 12), did you live in more than one town or city? Yes Is Not Selected

Q426 Consider your life between the ages of 6 and 12. How do you rate the quality of interracial relations in the neighborhood that you lived?

Answer If During your late childhood (age 6 to 12), did you live in more than one town or city? Yes Is Not Selected

Q427 Town or city you lived:

|  | 1 (1) | 2 (2) | 3 (3) | 4 (4) | 5 (5) | 6 (6) | 7 (7) |
| --- | --- | --- | --- | --- | --- | --- | --- |
| How do you rate the quality of interracial relations in the neighborhood that you lived? (1) |  |  |  |  |  |  |  |

Answer If During your early childhood (age 0 to 6), did you live in more than one town or city? Yes Is Selected

Q428 Consider your life between the ages of 6 and 12. How do you rate the quality of interracial relations in the neighborhoods that you lived? If you lived in more than one place, please report the average quality of interracial relations in all neighborhoods.

Answer If During your early childhood (age 0 to 6), did you live in more than one town or city? Yes Is Selected

Q429 Towns or cities you lived:

|  | 1 (1) | 2 (2) | 3 (3) | 4 (4) | 5 (5) | 6 (6) | 7 (7) |
| --- | --- | --- | --- | --- | --- | --- | --- |
| How do you rate the average quality of interracial relations in the neighborhoods that you lived? (1) |  |  |  |  |  |  |  |

Q430 Please click on the circle under the number that best corresponds with your answer to the question.

|  | 1 (1) | 2 (2) | 3 (3) | 4 (4) | 5 (5) | 6 (6) | 7 (7) |
| --- | --- | --- | --- | --- | --- | --- | --- |
| Based on your observation of individuals living in the same neighborhood as you, did people interact with others within their own race? (1) |  |  |  |  |  |  |  |

Q431 Please click on the circle under the number that best corresponds with your answer to the question.

|  | 1 (1) | 2 (2) | 3 (3) | 4 (4) | 5 (5) | 6 (6) | 7 (7) |
| --- | --- | --- | --- | --- | --- | --- | --- |
| Based on your observation of individuals living in the same neighborhood as you, did people interact with others outside their race? (1) |  |  |  |  |  |  |  |

Q432 Please click on the circle under the number that best corresponds with your answer to the question.

|  | 1 (1) | 2 (2) | 3 (3) | 4 (4) | 5 (5) | 6 (6) | 7 (7) |
| --- | --- | --- | --- | --- | --- | --- | --- |
| Racial segregation, refers to the separation of individuals by race, may be seen in schools, public transportations, and housing. How would you rate the racial segregation in the neighborhood that you lived? (1) |  |  |  |  |  |  |  |

Q159 Please think about your closest friends at this time (age 6 to 12), not acquaintances. Provide initials of between 5 and 20 closest friends (including relatives):

Q158 Please identify the race of those individuals

Q433 Please try to report as much as you can remember. If there is information that you do not remember, you may skip the question but please do not make up an answer.

Q434 Now consider your childhood between the ages of 13 and 18. This is roughly the time during which you were in middle school and high school. Please answer the following questions to the best of your ability.

Q435 1. Not including your family, what percentage of the adults you knew (friends, caretakers, neighbors) belonged to each of the following categories?

% Asian (1)

% Black (2)

% Latino (3)

% White (4)

% Other (5)

Q436 2. Not including your family, what percentage of the teens you knew (friends, classmates, neighbors) belonged to each of the following categories?

% Asian (1)

% Black (2)

% Latino (3)

% White (4)

% Other (5)

Q437 Please click on the circle under the number that best corresponds with your answer to the question.

|  | 1 (1) | 2 (2) | 3 (3) | 4 (4) | 5 (5) | 6 (6) | 7 (7) |
| --- | --- | --- | --- | --- | --- | --- | --- |
| How confident are you about the answers you gave for questions 1 & 2? (1) |  |  |  |  |  |  |  |

Q438 Please click on the circle under the number that best corresponds with your answer to the question.

|  | 1 (1) | 2 (2) | 3 (3) | 4 (4) | 5 (5) | 6 (6) | 7 (7) |
| --- | --- | --- | --- | --- | --- | --- | --- |
| How much would you say you remember about your childhood from age 13 to 18? (1) |  |  |  |  |  |  |  |

Q439 During your teenage years (age 13 to 18), did you live in more than one town or city?

- Yes (5)
- No (6)

Q440 In what town did you spend most of your teenage years (age 13 to 18)? If you lived in more than one place, please list the two places where you have spent the most time as accurately as you can:

Q441 Please describe the first town or city:

1st town or city: (1)

State (2)

ZIP code (3)

Answer If During your teenage years (age 13 to 18), did you live in more than one town or city? Yes Is Selected

Q442 Please describe the second town or city:

2nd town or city: (1)

State (2)

ZIP code (3)

Q443 Negative interracial relations refer to situations where the relationships among individuals from different races tend to be tense, and people tend to avoid going to areas where racial out-group members gather. Positive interracial relations refer to situations where interactions tend to be pleasant and harmonious.

Answer If During your teenage years (age 13 to 18), did you live in more than one town or city? Yes Is Not Selected

Q444 Consider your life between the ages of 13 and 18. How do you rate the quality of interracial relations in the neighborhood that you lived?

Answer If During your teenage years (age 13 to 18), did you live in more than one town or city? Yes Is Not Selected

Q445 Town or city you lived:

|  | 1 (1) | 2 (2) | 3 (3) | 4 (4) | 5 (5) | 6 (6) | 7 (7) |
| --- | --- | --- | --- | --- | --- | --- | --- |
| How do you rate the quality of interracial relations in the neighborhood that you lived? (1) |  |  |  |  |  |  |  |

Answer If During your teenage years (age 13 to 18), did you live in more than one town or city? Yes Is Selected

Q446 Consider your life between the ages of 13 and 18. How do you rate the quality of interracial relations in the neighborhoods that you lived? If you lived in more than one place, please report the average quality of interracial relations in all neighborhoods.

Answer If During your early childhood (age 0 to 6), did you live in more than one town or city? Yes Is Selected

Q447 Towns or cities you lived:

|  | 1 (1) | 2 (2) | 3 (3) | 4 (4) | 5 (5) | 6 (6) | 7 (7) |
| --- | --- | --- | --- | --- | --- | --- | --- |
| How do you rate the average quality of interracial relations in the neighborhoods that you lived? (1) |  |  |  |  |  |  |  |

Q448 Please click on the circle under the number that best corresponds with your answer to the question.

|  | 1 (1) | 2 (2) | 3 (3) | 4 (4) | 5 (5) | 6 (6) | 7 (7) |
| --- | --- | --- | --- | --- | --- | --- | --- |
| Based on your observation of individuals living in the same neighborhood as you, did people interact with others within their own race? (1) |  |  |  |  |  |  |  |

Q449 Please click on the circle under the number that best corresponds with your answer to the question.

|  | 1 (1) | 2 (2) | 3 (3) | 4 (4) | 5 (5) | 6 (6) | 7 (7) |
| --- | --- | --- | --- | --- | --- | --- | --- |
| Based on your observation of individuals living in the same neighborhood as you, did people interact with others outside their race? (1) |  |  |  |  |  |  |  |

Q450 Please click on the circle under the number that best corresponds with your answer to the question.

|  | 1 (1) | 2 (2) | 3 (3) | 4 (4) | 5 (5) | 6 (6) | 7 (7) |
| --- | --- | --- | --- | --- | --- | --- | --- |
| Racial segregation, refers to the separation of individuals by race, may be seen in schools, public transportations, and housing. How would you rate the racial segregation in the neighborhood that you lived? (1) |  |  |  |  |  |  |  |

Q160 Please think about your closest friends at this time (age 13 to 18), not acquaintances. Provide initials of between 5 and 20 closest friends (including relatives):

Q161 Please identify the race of those individuals

Q451 Please try to report as much as you can remember. If there is information that you do not remember, you may skip the question but please do not make up an answer.

Q452 Now consider your life now (i.e. at your current age). Please answer the following questions to the best of your ability.

Q453 1. Not including your family, what percentage of the adults you know (friends, caretakers, neighbors) belong to each of the following categories?

% Asian (1)

% Black (2)

% Latino (3)

% White (4)

% Other (5)

Q454 2. Not including your family, what percentage of the peers you know (friends, classmates, neighbors) belong to each of the following categories?

% Asian (1)

% Black (2)

% Latino (3)

% White (4)

% Other (5)

Q141 2.What percentage of individuals that you have dated belong to each of the following categories?

% Asian (1)

% Black (2)

% Latino (3)

% White (4)

% Other (5)

Q459 Where do you live?

town or city: (1)

State (2)

ZIP code (3)

Q461 Negative interracial relations refer to situations where the relationships among individuals from different races tend to be tense, and people tend to avoid going to areas where racial out-group members gather. Positive interracial relations refer to situations where interactions tend to be pleasant and harmonious.

Q462 Consider your life currently. How do you rate the quality of interracial relations in the neighborhood that you live in?

Q463 Town or city you live in:

|  | 1 (1) | 2 (2) | 3 (3) | 4 (4) | 5 (5) | 6 (6) | 7 (7) |
| --- | --- | --- | --- | --- | --- | --- | --- |
| How do you rate the quality of interracial relations in the neighborhood that you live in? (1) |  |  |  |  |  |  |  |

Q466 Please click on the circle under the number that best corresponds with your answer to the question.

|  | 1 (1) | 2 (2) | 3 (3) | 4 (4) | 5 (5) | 6 (6) | 7 (7) |
| --- | --- | --- | --- | --- | --- | --- | --- |
| Based on your observation of individuals living in the same neighborhood as you, do people interact with others within their own race? (1) |  |  |  |  |  |  |  |

Q467 Please click on the circle under the number that best corresponds with your answer to the question.

|  | 1 (1) | 2 (2) | 3 (3) | 4 (4) | 5 (5) | 6 (6) | 7 (7) |
| --- | --- | --- | --- | --- | --- | --- | --- |
| Based on your observation of individuals living in the same neighborhood as you, do people interact with others outside their race? (1) |  |  |  |  |  |  |  |

Q468 Please click on the circle under the number that best corresponds with your answer to the question.

|  | 1 (1) | 2 (2) | 3 (3) | 4 (4) | 5 (5) | 6 (6) | 7 (7) |
| --- | --- | --- | --- | --- | --- | --- | --- |
| Racial segregation, refers to the separation of individuals by race, may be seen in schools, public transportations, and housing. How would you rate the racial segregation in the neighborhood that you live in? (1) |  |  |  |  |  |  |  |

Q162 Please think about your closest friends now, not acquaintances. Provide initials of between 5 and 20 closest friends (including relatives):

Q163 Please identify the race of those individuals

Political What political party do you belong to or associate yourself with?

- Democrat (1)
- Republican (2)
- Independent (3)

School What type of high school did you attend?"

- General public (1)
- Specialized or magnet public (2)
- Private (3)
- Parochial or religious (4)

Age Age (in years)

Gender Gender

- Male (1)
- Female (2)
- Other: (3) ____________________

Q44 What is your native language?

Q142 Are you a United States citizen?

- Yes (1)
- No (2)

QCountry What country were you born in?

QYears If you were born in a country other than the United States, how long have you lived in the U.S (in years)?  If you were born in the United States, please go on to the next question.

Q66 What Ethnic group(s) do you identify with?

Q34 What religious group/groups do you identify with?

Q37 What was the name of your Kindergarten?

Name (1)

City/State (2)

Q38 What was the name of your Elementary School?

Name (1)

City/State (2)

Q39 What was the name of your Middle School?

Name (1)

City/State (2)

Q40 What was the name of your High School?

Name (1)

City/State (2)

Q69 Please click on the circle under the number that best corresponds with your answer to the question.

|  | 1 (1) | 2 (2) | 3 (3) | 4 (4) | 5 (5) | 6 (6) | 7 (7) |
| --- | --- | --- | --- | --- | --- | --- | --- |
| How conservative would you rate yourself? (1) |  |  |  |  |  |  |  |

Q70 Please click on the circle under the number that best corresponds with your answer to the question.

|  | 1 (1) | 2 (2) | 3 (3) | 4 (4) | 5 (5) | 6 (6) | 7 (7) |
| --- | --- | --- | --- | --- | --- | --- | --- |
| How liberal would you rate yourself? (1) |  |  |  |  |  |  |  |

Q145 Please click on the circle under the number that best corresponds with your answer to the question.

|  | 1 (1) | 2 (2) | 3 (3) | 4 (4) | 5 (5) | 6 (6) | 7 (7) |
| --- | --- | --- | --- | --- | --- | --- | --- |
| How strongly do you identify with other members of your ethnic group? (1) |  |  |  |  |  |  |  |

Q146 Please click on the circle under the number that best corresponds with your answer to the question.

|  | 1 (1) | 2 (2) | 3 (3) | 4 (4) | 5 (5) | 6 (6) | 7 (7) |
| --- | --- | --- | --- | --- | --- | --- | --- |
| How important is your ethnicity to your identity? (1) |  |  |  |  |  |  |  |

Q147 Please click on the circle under the number that best corresponds with your answer to the question.

|  | 1 (1) | 2 (2) | 3 (3) | 4 (4) | 5 (5) | 6 (6) | 7 (7) |
| --- | --- | --- | --- | --- | --- | --- | --- |
| How often do you think of yourself as a member of your ethnic group? (1) |  |  |  |  |  |  |  |

Q148 Please click on the circle under the number that best corresponds with your answer to the question.

|  | 1 (1) | 2 (2) | 3 (3) | 4 (4) | 5 (5) | 6 (6) | 7 (7) |
| --- | --- | --- | --- | --- | --- | --- | --- |
| How close do you feel to other members of your ethnic group? (1) |  |  |  |  |  |  |  |

SES How would you describe your family's social class position?

- Poor (1)
- Working Class (2)
- Middle Class (3)
- Upper Middle Class (4)
- Upper Class (5)

Education What is the highest level of education you have completed?

- No Formal Education (1)
- Elementary School (2)
- Some High School (3)
- Completed High School (4)
- Some College (5)
- BA/BS Degree (6)
- Some Graduate/Professional School (7)
- Hold Graduate/Professional Degree (8)

Q149


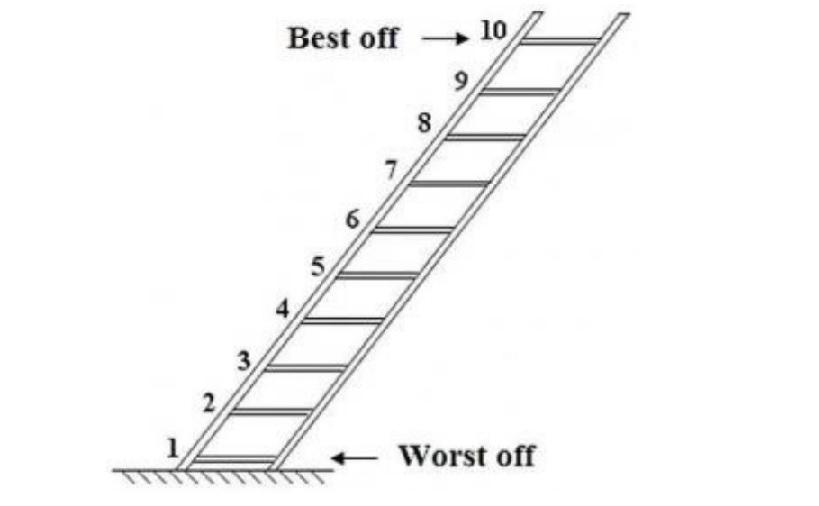


Q150 Think of this ladder as representing where people stand in their communities. People define community in different ways; please define it in whatever way is most meaningful to you. At the very top of the ladder are the people who have the highest standing in their community. At the very bottom are the people who have the lowest standing in their community.

Q151 Where would you place yourself on this ladder? Please select the step of the ladder you think you stand on at this time in your life, relative to other people in your community.

- Step 1 (1)
- Step 2 (2)
- Step 3 (3)
- Step 4 (4)
- Step 5 (5)
- Step 6 (6)
- Step 7 (7)
- Step 8 (8)
- Step 9 (9)
- Step 10 (10)

Q486


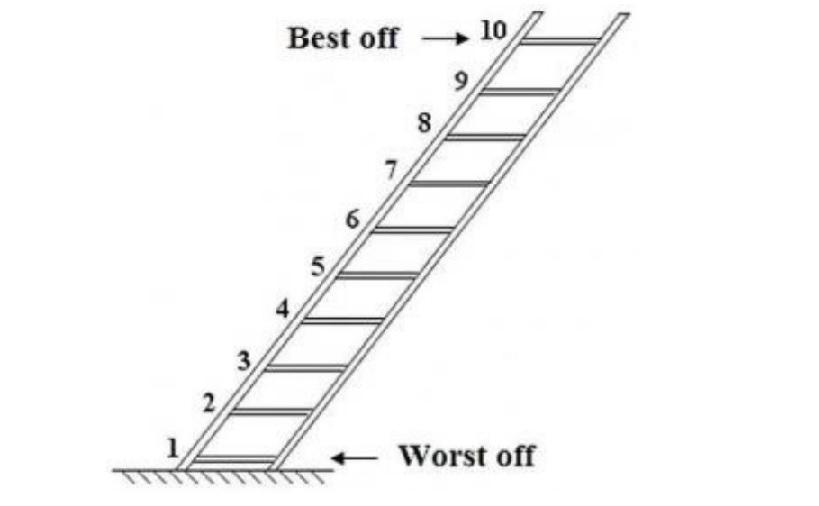


Q487 Think of this ladder as representing where people stand in the United States. At the top of the ladder are the people who are the best off -- those who have the most money, the most education, and the most respected jobs. At the bottom are the people who are the worst off -- who have the least money, least education, and the least respected jobs or no job. The higher up you are on this ladder, the closer you are to the people at the very top; the further down you are, the closer you are to the people at the very bottom.

Q488 Where would you place yourself on this ladder? Please select the step of the ladder you think you stand on at this time in your life, relative to other people in the United States.

- Step 1 (1)
- Step 2 (2)
- Step 3 (3)
- Step 4 (4)
- Step 5 (5)
- Step 6 (6)
- Step 7 (7)
- Step 8 (8)
- Step 9 (9)
- Step 10 (10)

Q155 What is the highest grade (or year) of regular school you have completed? (Select one.)

- Elementary School 01 (1)
- Elementary School 02 (2)
- Elementary School 03 (3)
- Elementary School 04 (4)
- Elementary School 05 (5)
- Elementary School 06 (6)
- Elementary School 07 (7)
- Elementary School 08 (8)
- High School 09 (9)
- High School 10 (10)
- High School 11 (11)
- High School 12 (12)
- College 13 (13)
- College 14 (14)
- College 15 (15)
- College 16 (16)
- Graduate School 17 (17)
- Graduate School 18 (18)
- Graduate School 19 (19)
- Graduate School 20+ (20)

Q156 What is the highest degree you earned?

- High school diploma or equivalency (GED) (1)
- Associate degree (junior college) (2)
- Bachelor's degree (3)
- Master's degree (4)
- Doctorate (5)
- Professional (MD, JD, DDS, etc.) (6)
- Other (please specify); (7) ____________________
- None of the above (less than high school) (8)

Q157 Which of the following best describes your current main daily activities and/ or responsibilities?

- Working full time (1)
- Working part-time (2)
- Unemployed or laid off (3)
- Looking for work (4)
- Keeping house or raising children full-time (5)
- Retired (6)

Q158 With regard to your current or most recent job activity

Q159 In what kind of business or industry do (did) you work? (For example: hospital, newspaper publishing, mail order house, auto engine manufacturing, breakfast cereal manufacturing.)

Q160 In what kind of work do (did) you do? (Job Title) (For example: registered nurse, personnel manager, supervisor of order department, gasoline engine assembler, grinder operator.)

Q161 How much did you earn, before taxes and other deductions, during the past 12 months?

- Less than $5,000 (1)
- $5,000 through $11,999 (2)
- $12,000 through $15,999 (3)
- $16,000 through $24,999 (4)
- $25,000 through $34,999 (5)
- $35,000 through $49,999 (6)
- $50,000 through $74,999 (7)
- $75,000 through $99,999 (8)
- $100,000 and greater (9)
- I don't know (10)
- No response (11)

Q162 How many people are currently living in your household, including yourself?

Number of people: (1)

Of these people, how many are children? (2)

Of these people, how many are adults? (3)

Of the adults, how many bring income into the household? (4)

Q163 Is the home where you live:

- Owned or being bought by you (or someone else in the household)? (1)
- Rented for money? (2)
- Occupied without payment of money or rent? (3)
- Other (specify): (4) ____________________

Q164 Which of these categories best describes your total combined family income for the past 12 months? This should include income (before taxes) from all sources, wages, rent from properties, social security, disability and/or veteran's benefits, unemployment benefits, workman's compensation, help from relatives (including child payments and alimony), and so on.

- Less than $5,000 (1)
- $5,000 through $11,999 (2)
- $12,000 through $15,999 (3)
- $16,000 through $24,999 (4)
- $25,000 through $34,999 (5)
- $35,000 through $49,999 (6)
- $50,000 through $74,999 (7)
- $75,000 through $99,999 (8)
- $100,000 and greater (9)
- I don't know (10)
- No response (11)

Q165 If you lost all your current source(s) of household income (your paycheck, public assistance, or other forms of income), how long could you continue to live at your current address and standard of living?

- Less than 1 month (1)
- 1 to 2 months (2)
- 3 to 6 months (3)
- 7 to 12 months (4)
- More than 1 year (5)

Q167 Suppose you needed money quickly, and you cashed in all of your (and your spouse's) checking and savings accounts, and any stocks and bonds. If you added up what you would get, about how much would this amount to?

- Less than $500 (1)
- $500 through $4,999 (2)
- $5,000 through $9,999 (3)
- $10,000 through $19,999 (4)
- $20,000 through $49,999 (5)
- $50,000 through $99,999 (6)
- $100,000 through $199,999 (7)
- $200,000 through $499,999 (8)
- $500,000 and greater (9)
- I don't know (10)
- No response (11)

Q168 If you now subtracted out any debt that you have (credit card debt, unpaid loans including car loans, home mortgage), about how much would you have left?

- Less than $500 (1)
- $500 through $4,999 (2)
- $5,000 through $9,999 (3)
- $10,000 through $19,999 (4)
- $20,000 through $49,999 (5)
- $50,000 through $99,999 (6)
- $100,000 through $199,999 (7)
- $200,000 through $499,999 (8)
- $500,000 and greater (9)
- I don't know (10)
- No response (11)

**Study 2 additional analyses**

**Implicit bias and interracial contact for all participants**

In the context of the model exploring the relationship between interracial childhood contact and implicit bias, IAT scores were significantly different from 0 when controlling for interracial childhood contact (b_o_=0.199, *t*(366)=7.935, *p*<0.001, *CI_lower_*=0.150 and *CI_upper_*=0.248). In the context of the model exploring the relationship between interracial current contact and implicit bias, IAT scores were significantly different from 0 when controlling for interracial current contact (b_o_=0.198, *t*(366)=7.811, *p*<0.001, *CI_lower_*=0.148 and *CI_upper_*=0.248).

Next, we regressed participant race (coded 1 for White and -1 for non-White), contact, and their interaction on implicit racial bias once for current contact and once for childhood contact because VIF factors were larger than 4 when both childhood and current contact were entered into the model simultaneously. When the interaction between participant race was separately assessed for current and childhood contact, participant race nor the interaction between race and contact emerged as significant predictors, indicating that the effects of contact on implicit racial bias were the same across racial groups (childhood contact model: b_o_=0.239, *t*(366)=5.100, *p*<0.001, *CI_lower_*=0.147 and *CI_upper_*=0.331, Child Contact b=-0.002, *t*(366)=-2.803, *p*=0.005, *partial r^2^*=0.021, *CI_lower_*=-0.003 and *CI_upper_*=-0.001, White versus Other Race b=0.059, *t*(366)=1.250, *p*=0.212, *partial r^2^*=0.004, *CI_lower_*=-0.034 and *CI_upper_*=0.151, Child Contact* White versus Other Race b=0.001, *t*(366)=0.788, *p*=0.431, *partial r^2^*=0.002, *CI_lower_*=-0.001 and *CI_upper_*=0.002; current contact model: b_o_=0.226, *t*(366)=5.537, *p*<0.001, *CI_lower_*=0.146 and *CI_upper_*=0.306, Current Contact b=-0.002, *t*(366)=-3.036, *p*=0.003, *partial r^2^*=0.025, *CI_lower_*=-0.003 and *CI_upper_*=-0.001, White versus Other Race b=0.065, *t*(366)=1.603, *p*=0.110, *partial r^2^*=0.007, *CI_lower_*=-0.015 and *CI_upper_*=0.145, Child Contact* White versus Other Race b=0.0003, *t*(366)=0.473, *p*=0.636, *partial r^2^*=.0007, *CI_lower_*=-0.001 and *CI_upper_*=0.001).

In the multiple regression model that included both childhood contact and current contact, IAT scores were significantly different from 0 when controlling for interracial current contact, interracial childhood contact, and their interaction (b_o_=0.189, *t*(366)=5.688, *p*<0.001, *CI_lower_*=0.123 and *CI_upper_*=0.254). Additionally, in the model where the interaction was removed, IAT scores were significantly different from 0 when controlling for interracial current contact and interracial childhood contact (b_o_=0.192, *t*(366)=7.570, *p*<0.001, *CI_lower_*=0.142 and *CI_upper_*=0.242).

When computing an overall average contact score across the lifespan, contact significantly predicted implicit bias (b=-0.003, *t*(366) =-6.979, *p*<0.001, *R^2^*=0.118, *CI_lower_*=-0.003 and *CI_upper_*=-0.002). Additionally, when controlling for overall contact, IAT scores remained significant, b_o_=0.195, *t*(366)=7.713, *p*<0.001), suggesting that there is a portion of unexplained variance in implicit attitudes not captured by Black and White contact across the lifespan.

**Implicit bias and interracial contact for non-black participants**

Although in the full model participant race did not interact with contact, we proceeded to verify the effects within our smaller samples of non-Black, White, and non-White participants. First, we explored the relationship between interracial contact and IAT for the non-Black sample. When comparing the participants’ IAT D-scores to 0, participants demonstrated pro-White implicit bias (*M_IATD_*=0.381, *SD*=.385, *t*(266)=16.16, *p*<0.001, *CI_lower_*=0.334 and *CI_upper_*=0.427).

We next explored whether participants had racial bias in childhood and current contact. Non-Black participants had significant racial bias in childhood contact when comparing scores to 0 (*M_childhood_*=-62.505%, *SD*=33.083%, *t*(266)=-30.872, *p*<0.001, *CI_lower_*=-66.491% and *CI_upper_*=-58.519%). Additionally, non-Black participants had significant racial bias in current contact when comparing scores to 0 (*M_current_*=-59.418%, *SD*=32.323%, *t*(266)=-30.037, *p*<0.001, *CI_lower_*=-63.313% and *CI_upper_*=-55.523%).

We then explored the relationship between interracial contact and implicit bias separately for childhood and current contact by regressing contact scores onto IATs. For childhood contact, linear regression results did not reveal a significant main effect of childhood contact, b=-0.001, *t*(266)=-1.777, *p*=0.071, *R^2^*=0.012, *CI_lower_*=-0.003 and *CI_upper_*=0.0001). In the context of the model exploring the relationship between interracial childhood contact and implicit bias, IAT scores were significantly different from 0 when controlling for interracial childhood contact (b_o_=0.302, *t*(266)=6.010, *p*<0.001, *CI_lower_*=0.203 and *CI_upper_*=0.400). Additionally, in this reduced sample, the effects of current contact did not emerge. Linear regression results did not reveal a significant main effect of current contact, b=-0.001, *t*(266)=-1.662, *p*=0.098, *R^2^* =0.010, *CI_lower_*=-0.003 and *CI_upper_*=0.0002). In the context of the model exploring the relationship between interracial current contact and implicit bias, IAT scores were significantly different from 0 when controlling for interracial current contact (b_o_=0.309, *t*(266)=6.278, *p*<0.001, *CI_lower_*=0.212 and *CI_upper_*=0.406).

To explore whether racial bias in childhood or current interracial contact has more explanatory power when predicting implicit racial bias, we regressed childhood contact, current contact, and their interaction on IATs. Because childhood contact and current contact are highly correlated (*r*(265)=0.783, *p*<0.001), we report variance inflation factors for each predictor in our analyses. Predictors were initially simultaneously entered into the regression model. Current contact (VIF=3.363), childhood contact (VIF=2.973), and their interaction (VIF=3.044) combined did not account for significant variance in implicit bias, (*r^2^_change_*=0.027, *F_change_*(3,263)=2.402, *p*=0.068). )^[[1]](#footnote-1)^. In the multiple regression model, there was a marginal interaction between current contact and childhood contact (b=0.00003, *t*(266)=1.957, *p*=0.051, *partial r^2^*=0.014, *CI_lower_*=-1.6097E-7 and *CI_upper_*=0.00005). No other predictors remained significant (Current Contact: b=0.001, *t*(266)=.560, *p*=0.576, *CI_lower_*=-0.002 and *CI_upper_*=0.003; Childhood Contact: b=-0.000004, *t*(266)=-0.004, *p*=0.997, *CI_lower_*=-0.002 and *CI_upper_*=0.002). IAT scores were significantly different from 0 when controlling for interracial current contact, interracial childhood contact, and their interaction (b_o_=0.305, *t*(266)=5.866, *p*<0.001, *CI_lower_*=0.203 and *CI_upper_*=0.408). Predictors were then entered into a stepwise regression from the most distal (childhood) to most proximal contact (current) followed by the interaction. In the stepwise regression, including current contact as a predictor in the model did not accounted for significant additional portion of variance (*r^2^ _change_*=0.001, *F_change_*(1,264)=.190, *p*=0.663). However, including the interaction accounted for a marginally significant additional portion of variance (*r^2^ _change_*=0.014, *F_change_*(1,263)=3.830, *p*=0.051).^[[2]](#footnote-2)^

For consistency, we also removed the interaction term and re-ran the analyses without the interaction term. When doing so, both the overall model (*r^2^_change_*=0.012, *F_change_*(2,264)=1.670, *p*=0.190) and the individual predictors did not emerge as significant (current contact b=-0.001, *t*(266)=-0.436, *p*=0.663, *partial r^2^*=0.0007, *CI_lower_*=-0.003 and *CI_upper_*=0.002, VIF=2.580 and childhood contact b=-0.001, *t*(266)=-0.763, *p*=0.446, *partial r^2^*=0.002, *CI_lower_*=-0.003 and *CI_upper_*=.001, VIF=2.580).

When computing an overall contact score across the lifespan, contact was not a significant predictor of implicit bias for the White participants (b=-0.001, *t*(266) =-1.829, *p*=0.068, *R^2^*=0.012, *CI_lower_*=-0.003 and *CI_upper_*=0.0001). Additionally, when controlling for overall contact, IAT scores remained significant, b_o_=0.277, *t*(266)=5.739, *p*<0.001, *CI_lower_*=0.195 and *CI_upper_*=0.398), suggesting there is a portion of unexplained variance in implicit attitudes for non-Black participants not captured by Black and White contact across the lifespan.

**Implicit bias and interracial contact for white participants**

We next explored the relationship between interracial contact and IAT in our White sample. First, we explored the relationship between interracial contact and IAT for the White sample. When comparing the participants’ IAT D-scores to 0, participants demonstrated pro-White implicit bias (*M_IATD_*=0.394, *SD*=.380, *t*(219)=15.389, *p*<0.001, *CI_lower_*=0.344 and *CI_upper_*=0.445).

We next explored whether participants had racial bias in childhood and current contact. White participants had significant racial bias in childhood contact when comparing scores to 0 (*M_childhood_*=-71.507%, *SD*=23.403%, *t*(219)=-45.320, *p*<0.001, *CI_lower_*=-74.616% and *CI_upper_*=-68.397%). Additionally, White participants had significant racial bias in current contact when comparing scores to 0 (*M_current_*=-65.942%, *SD*=25.740%, *t*(219)=-37.998, *p*<0.001, *CI_lower_*=-69.363% and *CI_upper_*=-62.522%).

We then explored the relationship between interracial contact and implicit bias separately for childhood and current contact by regressing contact scores onto IATs. For childhood contact, linear regression results did not reveal a significant main effect of childhood contact, b=-0.001, *t*(219)=-1.237, *p*=0.271, *R^2^*=0.007, *CI_lower_*=-0.004 and *CI_upper_*=0.001). In the context of the model exploring the relationship between interracial childhood contact and implicit bias, IAT scores were significantly different from 0 when controlling for interracial childhood contact (b_o_=0.297, *t*(219)=3.607, *p*<0.001, *CI_lower_*=0.135 and *CI_upper_*=0.460). Additionally, in this reduced sample, the effects of current contact did not emerge. Linear regression results did not reveal a significant main effect of current contact, b=-0.002, *t*(219)=-1.574, *p*=0.117, *R^2^* =0.011, *CI_lower_*=-0.004 and *CI_upper_*=0.0004). In the context of the model exploring the relationship between interracial current contact and implicit bias, IAT scores were significantly different from 0 when controlling for interracial current contact (b_o_=0.291, *t*(219)=4.136, *p*<0.001, *CI_lower_*=0.152 and *CI_upper_*=0.430).

To explore whether racial bias in childhood or current interracial contact has more explanatory power when predicting implicit racial bias, we regressed childhood contact, current contact, and their interaction on IATs. Because childhood contact and current contact are highly correlated (*r*(2189)=0.655, *p*<0.001), we report variance inflation factors for each predictor in our analyses. Predictors were initially simultaneously entered into the regression model. Current contact (VIF=10.388), childhood contact (VIF=5.977), and their interaction (VIF=21.777) combined did not account for significant variance in implicit bias, (*r^2^_change_*=0.020, *F_change_*(3,216)=1.482, *p*=0.220). Additionally, none of the predictors were significant except IAT scores controlling for the other factors (b_o_=0.472, *t*(219)=2.874, *p*=0.004). However, the VIF factors indicated multicollinearity. Therefore, we removed the interaction term and re-ran the analyses without the interaction term. When doing so, both the overall model (*r^2^_change_*=0.012, *F_change_*(2,217)=1.272, *p*=0.282) and the individual predictors did not emerge as significant (current contact b=-0.001, *t*(219)=-1.007, *p*=0.315, *partial r^2^*=0.005, *CI_lower_*=-0.004 and *CI_upper_*=0.001, VIF=1.750 and childhood contact b=-0.0004, *t*(219)=-0.276, *p*=0.783, *partial r^2^*=0.0004, *CI_lower_*=-0.003 and *CI_upper_*=.002, VIF=1.750). For consistency, we entered the predictors into a stepwise regression modeling first the most distal (childhood) to the most proximal contact (current) followed by the interaction. In the stepwise regression, including current contact as a predictor in the model did not account for a significant additional portion of variance (*r^2^ _change_*=0.005, *F_change_*(1,217)=1.014, *p*=0.315) nor did including the interaction (*r^2^ _change_*=0.009, *F_change_*(1,216)=1.891, *p*=0.171)^^[[3]](#footnote-3)^,^^[[4]](#footnote-4)^.

When computing an overall contact score across the lifespan, contact was not a significant predictor of implicit bias for the White participants (b=-0.002, *t*(219) =-1.461, *p*=0.146, *R^2^*=0.010, *CI_lower_*=-0.004 and *CI_upper_*=0.001). Additionally, when controlling for overall contact, IAT scores remained significant, b_o_=0.277, *t*(219)=3.274, *p*=0.001), suggesting there is a portion of unexplained variance in implicit attitudes for White participants not captured by Black and White contact across the lifespan.

**Implicit bias and interracial contact for non-white participants**

We next explored the relationship between interracial contact and IAT in our non-White sample. When comparing the participants’ IAT D-scores to 0, participants demonstrated pro-White implicit bias (*M_IATD_*=0.143, *SD*=0.444, *t*(146)=3.898, *p*<0.001, *CI_lower_*=0.070 and *CI_upper_*=0.215).

We next explored whether participants had racial bias in childhood and current contact. Non-White participants had significantly more contact with Blacks than Whites during childhood when comparing scores to 0 (*M_childhood_*=15.527%, *SD*=48.091%, *t*(146)=3.914, *p*<.001, *CI_lower_*=7.688% and *CI_upper_*=23.366%). However, non-White participants only had marginally significantly more contact with Blacks than Whites during childhood when comparing scores to 0 (*M_current_*=8.227%, *SD*=53.144%, *t*(146)=1.877, *p*=.063, *CI_lower_*=-.436% and *CI_upper_*=16.889%).

Next, we explored the relationship between interracial contact and implicit bias separately for childhood and current contact by regressing contact scores onto IATs. For childhood contact, linear regression results revealed a significant main effect of childhood contact, b=-0.002, *t*(146)=-3.266, *p*=0.001, *R^2^*=0.069, *CI_lower_*=-0.004 and *CI_upper_*=-0.001), such that more diversity in childhood contact related to lower implicit racial bias. In the context of the model exploring the relationship between interracial childhood contact and implicit bias, IAT scores were significantly different from 0 when controlling for interracial childhood contact (b_o_=0.180, *t*(146)=4.836, *p*<0.001, *CI_lower_*=0.107 and *CI_upper_*=0.254). Additionally, replicating previous work, linear regression results revealed a significant main effect of current contact, b=-0.002, *t*(146)=-3.197, *p*=0.002, *R^2^*=0.066, *CI_lower_*=-0.003 and *CI_upper_*=-0.001), such that more diversity in current contact related to lower implicit racial bias. In the context of the model exploring the relationship between interracial childhood contact and implicit bias, IAT scores were significantly different from 0 when controlling for interracial current contact (b_o_=0.160, *t*(146)=4.462, *p*<0.001, *CI_lower_*=0.089 and *CI_upper_*=0.231). Therefore, as childhood and current contact with Black relative to White individuals increases, implicit racial bias decreases.

To explore whether racial bias in childhood or current interracial contact has more explanatory power when predicting implicit racial bias, we regressed childhood contact, current contact, and their interaction on IATs. Current contact (VIF = 2.794), childhood contact (VIF = 2.617), and their interaction (VIF = 1.206) combined accounted for a small but significant 7.6% of variance in implicit bias, (*r^2^_change_*=0.076, *F_change_*(3,143)=3.904, *p*=0.010. In the context of the full model, none of the predictors were significant. Although the VIF factors did not indicate multicollinearity, childhood contact and current contact were still highly correlated (*r*(145)=0.786, *p*<.001). Additionally, we wanted to be consistent in our analysis approach across all of the samples; therefore, we removed the interaction term and re-ran the analyses. When doing so, the overall model was a significant fit (*r^2^_change_* =0.075, *F_change_*(2,144)=5.862, *p*=0.004), but not the individual predictors (current contact b=-0.001, *t*(146)=-1.026, *p*=0.307, *partial r^2^*=0.007, *CI_lower_*=-0.003 and *CI_upper_*=.001, VIF=2.616 and childhood contact b=-0.001, *t*(146)=-1.213, *p*=0.227, *partial r^2^*=0.010, *CI_lower_*=-0.004 and *CI_upper_*=.001, VIF=2.616). We also re-ran the stepwise regression for the non-White sample. In this stepwise regression, including current contact as a predictor in the model did not account for a significant additional portion of variance (*r^2^ _change_*=0.007, *F_change_*(1,144)=1.053, *p*=.307) nor did including the interaction (*r^2^ _change_*=0.0004, *F_change_*(1,143)=0.063, *p*=.802).^[[5]](#footnote-5)^

When computing an overall contact score across the lifespan, contact significantly predicted implicit bias (b=-0.003, *t*(146)=-3.349, *p*=0.001, *R^2^*=0.072, *CI_lower_*=-0.004 and *CI_upper_*=-0.001). Additionally, when controlling for overall contact, IAT scores remained significant, b_o_=0.177, *t*(146)=4.811, *p*<.001), suggesting that there is a portion of unexplained variance in implicit attitudes for non-White participants not captured by Black and White contact across the lifespan.

**Implicit bias and interracial contact for black participants**

Additionally, results for the Black only participants should be interpreted with the caveat that the reduced sample size reduced power. Although IAT scores were not on average different from 0, when accounting for childhood contact, IAT scores were significant, b_o_=0.122, *t*(99)=2.232, *p*=0.028) for Black participants. Similarly, when controlling for current contact, IAT scores were significant, b_o_=0.109, *t*(99)=2.232, *p*=0.028).

When computing an overall contact score across the lifespan, contact was a marginal predictor of implicit bias (b=-0.002, *t*(99)=-1.987, *p*=0.050, *R^2^*=0.039, *CI_lower_*= - 0.004 and *CI_upper_*=0.00002). Additionally, when controlling for current contact, IAT scores become significant, b_o_=0.127, *t*(99)=2.326, *p*=0.022), suggesting that there is a portion of unexplained variance in implicit attitudes for Black participants captured by Black and White contact across the lifespan.

To explore whether racial bias in childhood or current interracial contact has more explanatory power when predicting implicit racial bias, we regressed childhood contact, current contact, and their interaction on IATs. In the context of the full model, none of the predictors were significant, except the intercept (b_o_=0.130, *t*(99)=2.285, *p*=0.025).

**Implicit bias and interracial contact for first wave of data collection**

To explore whether data collection wave had an impact on the findings, we included a predictor for wave (First =-1, Second =1) for the whole sample (n=367) in separate regressions for childhood contact and current contact. In these analyses, wave did not interact with contact (Childhood Contact b=-0.003, *t*(366)=-3.350, *p*=0.001, *partial r^2^*=0.030, *CI_lower_*=-0.005 and *CI_upper_*=-0.001, Wave b=0.050, *t*(366)=0.688, *p*=0.492, *partial r^2^*=0.001, *CI_lower_*=-0.093 and *CI_upper_*=0.192, Childhood Contact* Wave b=0.001, *t*(366)=0.566, *p*=0.571, *partial r^2^*=0.0009, *CI_lower_*=-0.001 and *CI_upper_*=0.002; Current Contact b=-0.002, *t*(366)=-2.513, *p*=0.012, *partial r^2^*=0.017, *CI_lower_*=-0.004 and *CI_upper_*=-0.0005, Wave b=-0.034, *t*(366)=-0.533, *p*=0.594, *partial r^2^*=0.0008, *CI_lower_*=-0.157 and *CI_upper_*=0.090, Childhood Contact* Wave b=-0.001, *t*(366)=-0.602, *p*=0.548, *partial r^2^*=0.001, *CI_lower_*=-0.002 and *CI_upper_*=0.001). These results indicate that the effects of Study 2 do not depend on the data collection wave.

Although the effects did not depend on data collection wave because the two waves of data collection were combined in a posthoc fashion, we also wanted to present the results for each wave separately for interested readers. Initially, one hundred participants were recruited for a master’s thesis via Amazon’s Mechanical Turk (MTurk) and compensated $4. All U.S.-based workers at least 18 years of age with more than a 65% approval rating were eligible to participate. The final sample included 90 White participants (50 females; *M*_age_ = 37.57 years, *SD_age_* = 11.917 years, *Min_age_* = 20 years, *Max_age_* = 72 years). There were 31 democrats, 35 republicans, 24 independents, and 83.3% identified as middle class or lower. Please interpret the findings with caution given the small sample size in each wave.

When comparing the participants’ IAT D-scores to 0, participants demonstrated pro-White implicit bias (*M_IATD_*= 0.377, *SD*=0.369, *t*(89)=9.699, *p*<0.001, *CI_lower_*=0.300 and *CI_upper_*=0.455).

We next explored whether participants had racial bias in childhood and current contact. Participants had significant racial bias in childhood contact when comparing scores to 0, indicating less childhood contact with Blacks than Whites (*M_childhood_*= -74.815%, *SD*=23.349%, *t*(89)=-30.397, *p*<0.001, *CI_lower_*=-79.705% and *CI_upper_*=-69.924%). Participants also had significant racial bias in current contact when comparing scores to 0, indicating less current contact with Blacks than Whites (*M_current_*= -68.382%, *SD*=25.251%, *t*(89)=-25.691, *p*<0.001, *CI_lower_*=-73.670% and *CI_upper_*=-63.093%).

Next, we explored the relationship between interracial contact and implicit bias separately for childhood and current contact by regressing contact scores onto IATs. For childhood contact, linear regression results revealed a significant main effect of childhood contact, b=-0.004, *t*(89)=-2.233, *p*=0.028, *R^2^* = 0.054, *CI_lower_*=-0.007 and *CI_upper_*=-0.0004, such that greater diversity in childhood contact related to less implicit racial bias. In this smaller sample, results did not reveal a significant main effect of current contact, b=-0.002, *t*(89)=-1.076, *p*=0.285, *R^2^* = 0.013, *CI_lower_*=-0.005 and *CI_upper_*=0.001.

**Implicit bias and interracial contact for second wave of data collection**

Three hundred additional participants were recruited to increase the diversity of the final sample via Amazon’s Mechanical Turk (MTurk) and compensated $4. All U.S.-based workers at least 18 years of age with more than a 65% approval rating were eligible to participate. The final sample from this wave included 277 participants (155 females, 121 male, 1 did not report; *M*_age_ = 32.55 years, *SD_age_* = 9.928 years, *Min_age_* = 18 years, *Max_age_* = 64 years, 1 did not report). There were 142 democrats, 30 republicans, 103 independents, and 87.6% identified as middle class or lower. Please interpret the findings with caution given the reduced sample size in each wave.

When comparing the participants’ IAT D-scores to 0, participants demonstrated pro-White implicit bias (*M_IATD_*= 0.266, *SD*=0.438, *t*(276)=10.112, *p*<0.001, *CI_lower_*=0.215 and *CI_upper_*=0.318).

We next explored whether participants had racial bias in childhood and current contact. Participants had significant racial bias in childhood contact when comparing scores to 0, indicating less childhood contact with Blacks than Whites (*M_childhood_*= -24.245%, *SD*=57.195%, *t*(276)=-7.055, *p*<0.001, *CI_lower_*=-31.010% and *CI_upper_*=-17.480%). Participants also had significant racial bias in current contact when comparing scores to 0, indicating less current contact with Blacks than Whites (*M_current_*= -25.790%, *SD*=55.893%, *t*(276)=-7.679, *p*<0.001, *CI_lower_*=-32.401% and *CI_upper_*=-19.178%).

Next, we explored the relationship between interracial contact and implicit bias separately for childhood and current contact by regressing contact scores onto IATs. For childhood contact, linear regression results revealed a significant main effect of childhood contact, b=-0.003, *t*(276)=-5.985, *p*<0.001, *R^2^* = 0.115, *CI_lower_*=-0.003 and *CI_upper_*=-0.002, such that greater diversity in childhood contact related to less implicit racial bias. Results revealed a significant main effect of current contact, b=-0.003, *t*(276)=-6.115, *p*<0.001, *R^2^* = 0.120, *CI_lower_*=-0.004 and *CI_upper_*=-0.002), such that greater diversity in current contact related to less implicit racial bias.

**Additional general discussion considerations**

The IAT, while being extremely reliable and versatile, also poses some distinct challenges. Due to the nature of the two category IAT, it is difficult to disentangle the contribution of bias for or against any group without some reference group. Hence, it is possible for a participant to obtain D-scores that indicate that they have a bias for White Americans, when in fact they have positive associations for people of both races with stronger positive associations for White Americans (Allport, 1954; Brewer, 1999). In addition, contact may shape associations to one group more than another group. Future research should consider using a single category IAT to explore non-comparative associations.

Although contact correlates with implicit biases, the effects were small and IAT scores were significant when accounting for contact. Therefore, there remains an amount of variance in implicit racial bias not accounted for by contact. Moreover, because childhood and current contact are highly correlated it is difficult to disentangle the contribution of each to reducing implicit racial bias. Previous research has found that White children develop racial biases that are just as strong as implicit racial biases observed in adults (Newheiser & Olson, 2012), perhaps implying that implicit associations develop early and remain pervasive. In the current research, although some of the relationship between childhood contact and implicit racial bias could be explained by current contact, childhood contact remained a marginally significant predictor when including current contact and the interaction between childhood contact and current contact across all participants. In addition, adding childhood contact accounted for a marginally significant change in fit across all participants. This may imply that childhood contact is a slightly stronger predictor of implicit racial bias than current contact and future research should further explore this possibility. However, for Black participants, current contact seemed to be the more important predictor of implicit racial bias and childhood contact did not significantly account for additional variance. Future research should explore the differential impact of current and childhood contact on implicit associations across racial groups.

The current research also does not disentangle the contributions of quality of contact from quantity of contact (e.g., Jelenec & Steffens, 2002; Teachman & Brownell, 2001). Although participants reported the amount of contact with Blacks and Whites, it could be the case that a particular kind of contact was driving the effects. For example, contact quantity has been shown to be unrelated to implicit bias across other social categories (Jelenec & Steffens, 2002; Teachman & Brownell, 2001). However, Prestwich, Kenworthy, Wilson, & Kwan-Tat (2008), found that contact quality influenced explicit attitudes, while contact quantity influenced implicit attitudes. In a recent meta-analysis exposure to outgroup members—regardless of the quality of that experience—resulted in more positive outgroup attitudes (Pettigrew & Tropp, 2006). Future research should explore how quality versus quantity of contact shapes implicit associations across the lifespan.

**References**

Aberson, C. L., & Haag, S. C. (2007). Contact, perspective taking, and anxiety as predictors of stereotype endorsement, explicit attitudes, and implicit attitudes. *Group Processes & Intergroup Relations, 10*(2), 179-201.

Allport, G. (1954). *The nature of prejudice.* Reading, MA: Addison-Wesley.

Berinsky, A. J., Huber, G. A., & Lenz, G. S. (2012). Evaluating online labor markets for experimental research: Amazon.com's Mechanical Turk. *Political Analysis*, *20*(3), 351-368.

Brewer, M. B. (1999). The psychology of prejudice: In-group love or out-group hate?

*Journal of Social Issues*, *55*(3), 429-444.

Buhrmester, M., Kwang, T., & Gosling, S. D. (2011). Amazon's Mechanical Turk a new source of inexpensive, yet high-quality, data? *Perspectives on Psychological Science*, *6*(1), 3-5.

Cloutier, J., Li, T., & Correll, J. (2014). The impact of childhood experience on amygdala

response to perceptually familiar Black and White faces. *Journal of Cognitive*

*Neuroscience, 26*(9), 1992-2004.

Cloutier, J.,  Li, T., Mišić, B., Correll, J., & Berman, M. (in press). Brain network activity during face perception: The impact of perceptual familiarity and individual differences in childhood experience. *Cerebral Cortex*.

Crump M. J. C., McDonnell J. V., Gureckis T. M. (2013). Evaluating Amazon's Mechanical Turk as a tool for experimental behavioral research. *PLoS ONE 8*(3), e57410. 10.1371/j.0057410

Horton, J. J., Rand, D. G., & Zeckhauser, R. J. (2011). The online laboratory: Conducting experiments in a real labor market. *Experimental Economics, 14*(3), 399-425.

Faul, F., Erdfelder, E., Lang, A.-G., & Buchner, A. (2007). G*Power 3: A flexible

statistical power analysis program for the social, behavioral, and biomedical sciences. *Behavior Research Methods*, *39*, 175-191.

Faul, F., Erdfelder, E., Buchner, A., & Lang, A.-G. (2009). Statistical power analyses using G*Power 3.1: Tests for correlation and regression analyses. *Behavior Research Methods*, *41*, 1149-1160.

Jelenec, P., & Steffens, M. C. (2002). Implicit attitudes toward elderly women and men. *Current Research in Social Psychology*, *7*(16), 275-293.

Mason, W., & Suri, S. (2012). Conducting behavioral research on Amazon’s Mechanical Turk. *Behavior Research Methods*, *44*(1), 1-23.

Newheiser, A. K., & Olson, K. R. (2012). White and Black American children’s implicit

intergroup bias. *Journal of Experimental Social Psychology, 48*(1), 264-270.

Paolacci, G., Chandler, J., & Ipeirotis, P. G. (2010). Running experiments on amazon mechanical turk. *Judgment and Decision Making*, *5*(5), 411-419.

Pettigrew, T. F., & Tropp, L. R. (2006). A meta-analytic test of intergroup contact theory*. Journal of Personality and Social Psychology*, *90*(5), 751-783.

Prestwich, A., Kenworthy, J. B., Wilson, M., & Kwan‐Tat, N. (2008). Differential relations between two types of contact and implicit and explicit racial attitudes. *British Journal of Social Psychology*, *47*(4), 575-588.

Rand, D. G. (2012). The promise of Mechanical Turk: How online labor markets can help theorists run behavioral experiments. *Journal of Theoretical Biology*, *299*, 172-179.

Teachman, B. A., & Brownell, K. D. (2001). Implicit anti-fat bias among health professionals: is anyone immune?. *International Journal of Obesity & Related Metabolic Disorders*, *25*(10).

1. Inspection of the normal P-P plot and a scatterplot of the standardized residuals did not reveal normality violations. [↑](#footnote-ref-1)
2. If instead the most proximal predictor is added first (current contact) into a stepwise regression, including childhood contact as a predictor in the model accounts for a marginally significant additional portion of variance (*r^2^_change_*=0.002, *F_change_*(1,264)=.583, *p*=0.446). [↑](#footnote-ref-2)
3. The interaction change statistics should be considered in light of a high degree of multicollinearity between current and childhood contact for this sub-sample. [↑](#footnote-ref-3)
4. If instead the most proximal predictor is added first (current contact) into the stepwise regression model, including childhood contact as a predictor in the model does not account for a significant additional portion of variance (*r^2^_change_* = 0.0003, *F_change_*(1,217)=0.076, *p*=.783) in this sample. [↑](#footnote-ref-4)
5. If instead the most proximal predictor is added first (current contact) into the stepwise regression model, including childhood contact as a predictor in the model does not account for a significant additional portion of variance (*r^2^ _change_*=0.009, *F_change_*(1,144)=1.472, *p*=.227) in this sample. [↑](#footnote-ref-5)
